# Transmission blocking sugar baits for the control of *Leishmania* development inside sand flies using environmentally friendly beta-glycosides and their aglycones

**DOI:** 10.1186/s13071-018-3122-z

**Published:** 2018-11-30

**Authors:** Tainá Neves Ferreira, Daniela Pita-Pereira, Samara Graciane Costa, Reginaldo Peçanha Brazil, Caroline Silva Moraes, Hector Manuel Díaz-Albiter, Fernando Ariel Genta

**Affiliations:** 1 Laboratório de Bioquímica e Fisiologia de Insetos, Instituto Oswaldo Cruz, FIOCRUZ, Rio de Janeiro, Brazil; 2 Laboratório de Biologia Molecular de Doenças Endêmicas, Instituto Oswaldo Cruz, FIOCRUZ, Rio de Janeiro, Brazil; 3 Laboratório de Doenças Parasitárias, Instituto Oswaldo Cruz, FIOCRUZ, Rio de Janeiro, Brazil; 4 Instituto Nacional de Ciência e Tecnologia em Entomologia Molecular, Rio de Janeiro, Brazil; 5 Wellcome Centre for Molecular Parasitology, University of Glasgow, Glasgow, UK; 6 El Colegio de la Frontera Sur (ECOSUR), Unidad Villahermosa, Villahermosa, México

**Keywords:** *Lutzomyia longipalpis*, *Leishmania*, Longevity, Beta-glycosides, Trehalase, Beta-glucosidase, Sugar bait

## Abstract

**Background:**

The sand fly *Lutzomyia longipalpis* is the main vector of American visceral leishmaniasis, a disease caused by parasites of the genus *Leishmania*. Adults of this insect feed on blood (females only) or sugar from plant sources, but their digestion of carbohydrates is poorly studied. Beta-glycosides as esculin and amygdalin are plant compounds and release toxic compounds as esculetin and mandelonitrile when hydrolyzed. Beta-glucosidase and trehalase are essential enzymes in sand fly metabolism and participate in sugar digestion. It is therefore possible that the toxic portions of these glycosides, released during digestion, affect sand fly physiology and the development of *Leishmania*.

**Results:**

We tested the oral administration to sand flies of amygdalin, esculin, mandelonitrile, and esculetin in the sugar meal. These compounds significantly decreased the longevity of *Lutzomyia longipalpis* females and males. *Lutzomyia longipalpis* adults have significant hydrolytic activities against esculin and feeding on this compound cause changes in trehalase and β-glucosidase activities. Female trehalase activity is inhibited *in vitro* by esculin. Esculin is naturally fluorescent, so its ingestion may be detected and quantified in whole insects or tissue samples stored in methanol. Mandelonitrile neither affected the amount of sugar ingested by sand flies nor showed repellent activity. Our results show that mandelonitrile significantly reduces the viability of *L. amazonensis*, *L. braziliensis*, *L. infantum* and *L. mexicana*, in a concentration-dependent manner. Esculetin caused a similar effect, reducing the number of *L. infantum* and *L. mexicana*. Female *L. longipalpis* fed on mandelonitrile had a reduction in the number of parasites and prevalence of infection after seven days of infection with *L. mexicana*, either by counting in a Neubauer chamber or by qPCR assays.

**Conclusions:**

Glycosides have significant effects on *L. longipalpis* longevity and metabolism and also affect the development of parasites in culture and inside the insect. These observations might help to conceptualize new vector control strategies using transmission blocking sugar baits.

**Electronic supplementary material:**

The online version of this article (10.1186/s13071-018-3122-z) contains supplementary material, which is available to authorized users.

## Background

Leishmaniasis is a group of diseases caused by protozoan parasites belonging to the genus *Leishmania*. These parasites are transmitted to humans by the bites of female phlebotomine sand flies. Based on different clinical manifestations, there are three main forms of leishmaniasis: cutaneous (CL), mucocutaneous (MCL) and kala-azar or visceral (VL) [1, 2].

These diseases are classified as neglected tropical diseases (NTD) and endemic in 98 countries worldwide. Leishmaniasis is strongly associated with poverty, but environmental and climatologic factors also influence its epidemiology. Recent studies by the Pan American Health Organization (PAHO) have estimated that ~75% of CL cases occur in ten countries, four of which belong to the American continent (Brazil, Colombia, Peru and Nicaragua), and ~90% of VL cases are found in Brazil, Ethiopia, India, Bangladesh and Sudan [1, 3].

*Leishmania* parasites have part of their life-cycle occurring in the sand fly. Since they develop entirely in the digestive system of the vector, they probably interact with digestive enzymes and other structures from the intestinal tract of the vector [4]. Research groups have described different compounds with anti-*Leishmania* action, mainly from natural sources such as furoquinolines and coumarins [5], naphthoquinone-derived products [6] and chalcones, saponins and alkaloids [7]. However, these studies have focused on their effect against parasites *in vitro* or inside the mammalian host, while their effect during *Leishmania*-sand fly interactions remains unknown.

Adult female sand flies feed on blood, and both sexes feed on plant sugars like nectar, honeydew or fruits. Sugar acts as an energy source and affects the development of parasites inside the insect’s digestive tract. Parasites can capture sugars from the gut contents, having specialized enzymes for this [8–11]. Plant-feeding preferences in sand flies and mosquitoes have been described in Old World species [12–16], and the possible use of sugar baits with insecticides has been proposed for the control of these insects [17, 18].

Vector control is one of the key strategies to reduce the number of leishmaniasis cases, which needs more research and development [19]. Recently in the Americas, some strategies are combining vector control with interference against reservoirs (dog collars, culling, vaccination) or revised human treatment. Promising results in leishmaniasis control are expected with the concomitant use of insecticide indoor residual spraying, interventions at vector breeding sites, prevention methods (repellent use), environmental management, and health education and social-based strategies [20–22].

However, in many cases the current vector control actions are still based on the sole use of chemical insecticides like organochlorines (DDT), organophosphates (malathion), carbamates and synthetic pyrethroids (cypermethrin, deltamethrin, among others). The indiscriminate use of this strategy poses risks related to environmental and human contamination [22].

**Fig. 1:**
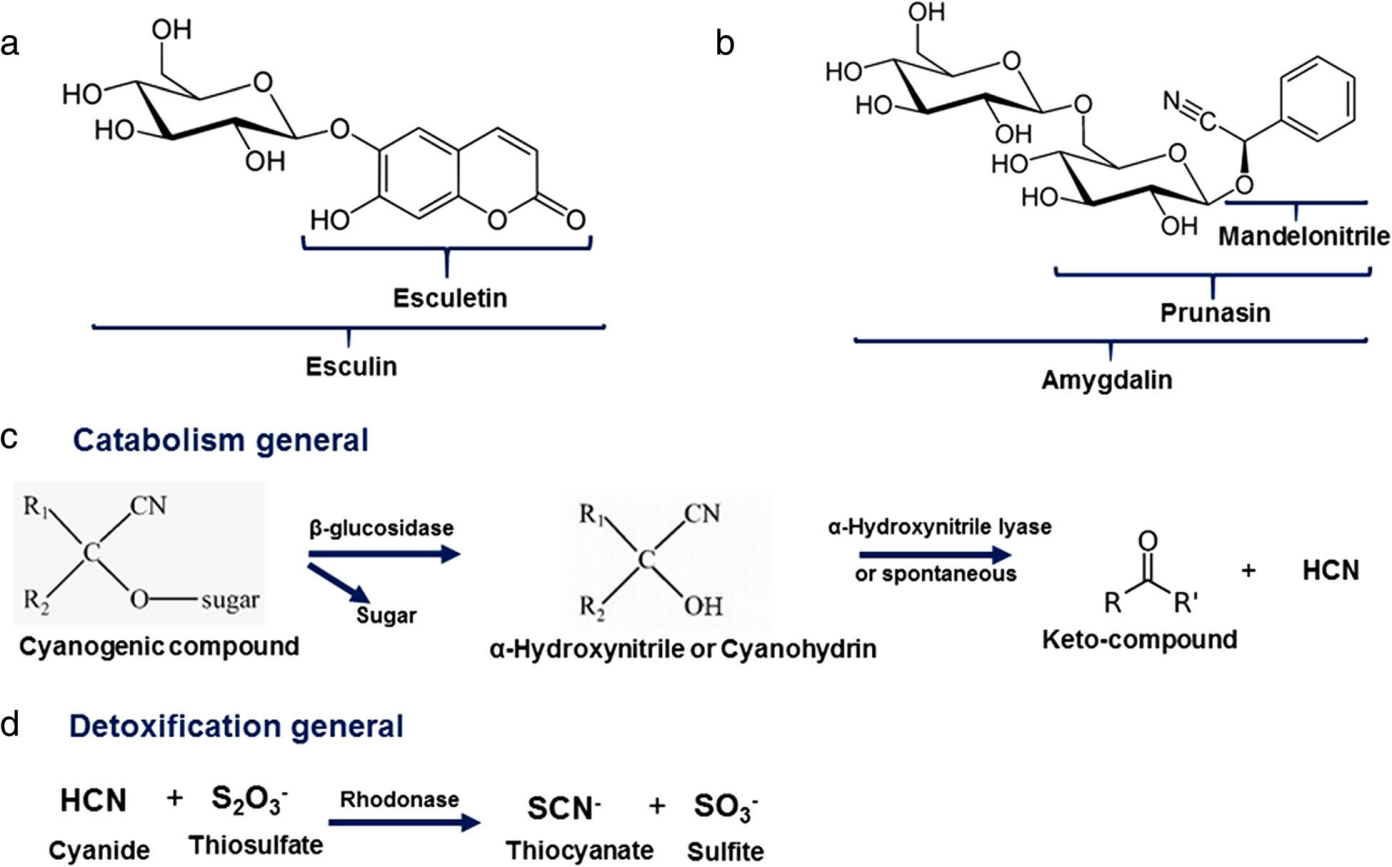
Glycosides, aglycones and catabolism and detoxification reactions in cyanogenic compounds in different organisms (**c** and **d** were made according literature information [61]). **a** Esculin and esculetin molecular structural formula. **b** Mandelonitrile, prunasin and amigdalyn molecular structural formula. **c** Catabolism reactions. **d** Detoxification reaction

β-glucosidases [E.C.3.2.1.21] are a group of enzymes that catalyze the hydrolysis of glycosidic bonds, with the release of non-reducing terminal glucose residues from oligosaccharides, alkyl or aryl β-D-glycosides [23]. β-glycosides play an essential role in the relationship between plants and insects as they help to avoid insect herbivory in plants that produce toxic glycosides, which may correspond to 1% of the plant dry weight. Many of these plant glycosides have hydrophobic aglycones (non-glycidic moieties), and after hydrolysis by insect intestinal β-glucosidases, they are released as harmful molecules, such as cyanide or alkylating agents (Fig. 1) [24]. As a defense against toxic glycosides from plants, some insects regulate the activity of intestinal β-glucosidase without affecting the final digestion of other substances, which are hydrolyzed by different enzymes [24].

Mandelonitrile and esculetin are well-known examples of toxic aglycones, which are released after hydrolysis of the glycosides amygdalin and esculin, respectively (Fig. 1a, b). These glycosides are commonly found in nature. Amygdalin, for example, is part of the composition of apricots (*Prunus armeniaca*), seeds, roots and leaves of sweet and bitter almonds (*Prunus dulcis*), peaches (*Prunus persica*) and plums (*Prunus domestica*) [25]. Esculin is found in the genus *Aesculus*, especially in chestnuts, and in fruits such as Japanese bitter orange (*Aegle marmelos*), blueberry (*Vaccinium myrtillus*) and wildberry (subgenus *Eubatus*). Esculin is also found in seeds (tonka bean *Dipteryx odorata*), leaves (*Murraya paniculata*) and roots (*Ferulago campestris*) [26].

Moreover, it has been already demonstrated that the inhibition of trehalases by these glycosides is a possible mechanism of toxicity of these compounds for insects [27]. Trehalases [E.C.3.2.1.28] are central enzymes in the metabolism of carbohydrates in insects because trehalose (alpha-1,1-glucopyranosyl-glucose) is the principal circulating sugar in the hemolymph of these invertebrates [24, 27]. Trehalase is necessary for the uptake of glucose by virtually all insect cells, so its inhibition is severely detrimental in these organisms [24, 27].

Glucosidases have a significant role in sand fly physiology and the establishment of interactions with *Leishmania* species, mainly α-glucosidases [28, 29]. Despite previous studies [28–31], little is known about the effect of plant-derived secondary metabolites during the interaction between parasites and vector, or even on basic sand fly digestive physiology. In this paper, we describe the effect of β-glycosides and their aglycones on longevity, β-glucosidase and trehalase activities in adult *L. longipalpis*. We also recorded the anti-*Leishmania* activity of these compounds and the effect of mandelonitrile in the *in vivo* reduction of infection of sand flies by *L. mexicana*. Additionally, we showed that compounds as esculin and mandelonitrile might block the transmission of leishmaniasis with no repellent effects or reduction in the amount of sugar ingested. In this way, these compounds might be promising tools for starting the development of anti-parasitic sugar baits with less selective pressure for resistance in the vector populations.

## Methods

### Longevity curves

All insects used in this work came from a colony first started ~20 years ago from individuals from Jacobina, Brazil, and were reared under standard laboratory conditions [32]. Twenty female or male sand flies were placed in 13 × 13 × 13 cm mesh cubic cages inside a plastic bag. Humidity was regulated by adding 1 ml of distilled water to a piece of paper towel inside the cage daily. Insects were offered the experimental compounds diluted in a 70% w/v sucrose solution. Two milliliters of each solution was added to a 0.2 g cotton pad and offered *ad libitum*. The final concentration of the compounds was 0.1% w/v according to Silva et al. [27]. Seventy percent w/v sucrose solution was offered as a negative control. Sucrose solutions were made using refined commercial sugar and autoclaved before addition of experimental compounds. Insects were kept inside a BOD-type incubator (26 ± 1 °C, 50–70% humidity). Insects were monitored daily, and mortality was recorded every day. Cotton pads and solutions were changed every 2–3 days. Amygdalin (cat. no. A6005), esculin (cat. no. E8250), esculetin (cat. no. 246573) and mandelonitrile (cat. no. 116025) were from Sigma-Aldrich (St. Louis, USA). Before adding to the sucrose solution, 1% (w/v) stock solutions of esculetin were prepared in 10% NaOH. Controls for experiments with esculetin included 10% NaOH in the same proportions. Final concentrations in the sugar meal are 2.6 mM esculin, 2.2 mM amygdalin, 7.5 mM mandelonitrile and 5.6 mM esculetin.

### Trehalase and β-glucosidase assays and *in vitro* or *in vivo* effects of test compounds in enzyme activities

For all enzyme activity assays, two types of experiments were performed: *in vitro*, where the test compound was added to the assay mixture with the substrate corresponding to the enzymatic assay, and *in vivo*, in which the insects were previously fed on sucrose solution containing the test compound. For *in vitro* experiments, adult insects were collected upon emergence, sorted out by sex and fed on a sucrose 70% (w/v) solution *ad libitum* for 2–5 days. For *in vivo* assays, adults were collected and maintained on 70% (w/v) sucrose solution with 0.1% (w/v) test compound *ad libitum* for seven days. The negative control was fed on a standard 70% (w/v) sucrose solution.

In all groups, insects were dissected by separating the midgut from the rest of the body [28]. Tissues were homogenized with a mechanical pellet pestle (Sigma-Aldrich) in 200 mM sodium citrate buffer (pH 6) supplemented with protease inhibitors 20 mM (final concentration in samples) phenylmethylsulfonyl fluoride (PMSF), 20 μM (final concentration in samples) pepstatin A, and 20 μM (final concentration in samples) E-64 (N-[*trans*-epoxysuccinyl]-L-leucine 4-guanidinobutylamide). Tissue-buffer proportions were 1:20 μl for trehalase and 1:6 μl for β-glucosidase assays.

Homogenates were centrifuged at 20,000× *g* for 5 min at 4 °C. Supernatants were collected and named as gut contents or rest of body soluble fraction. Pellets were resuspended in the original volume of fresh sodium citrate buffer and named gut tissue or rest of body tissues. In enzyme assays, 10 μl of the sample and 10 μl of substrate (10 mM trehalose or cellobiose) with or without 0.2% (w/v) test compound (esculin, amygdalin, esculetin or mandelonitrile) in aqueous solution were mixed in either 96-well conical bottom plates or 200 μl microtubes. After mixing with substrate, protease inhibitors concentration was reduced to 10 mM and 10 μM.

Reactions were stopped after different times of incubation at 30 °C by incubating at 100 °C for 3 min [33]. Points were withdrawn at intervals of 15 min for trehalase and 1 h for β-glucosidase, with total assay times of 75 min or 4 h, respectively. After interruption, 200 μl of TGO reagent (Glucose Colorimetric Assay Kit, ref. K082, Bioclin, Belo Horizonte, Brazil) were added, and the samples were kept at 37 °C for 30 min. Absorbance was read at 550 nm in a 96-well plate. This assay was adapted from [23, 34]. One enzyme unit (U) corresponds to the amount of enzyme that hydrolyzes 1 μmol of substrate molecules per minute. Assays were performed in conditions where product release was proportional to time and protein content. Controls without substrate or sample were incubated and revealed in the same conditions.

To assays with the presence of esculetin, 20 μl of 50 mM K_2_Cr_2_O_7_ were added after interruption, and the reactions were incubated for 30 min at 37 °C before the addition of TGO. This step was done because esculetin interferes with glucose detection. For the preparation of esculetin solutions for the assays, we used stock solutions in 10% NaOH. The final concentration of NaOH in the assay mixture was kept at 10 mM, and controls included the same concentration of NaOH.

### Esculin hydrolysis assay

To register the hydrolysis of esculin, 0.2% (w/v) esculin aqueous solution was used as a substrate. As described above, for the *in vitro* assays, insects were maintained on a 70% sucrose solution diet. In a separate *in vivo* experiment, experimental flies were fed after emergence on a 70% sucrose solution supplemented with 0.1% (w/v) esculin. Insects were dissected, tissues were homogenized, and assays were performed in the conditions described above for trehalase, using esculin 0.2% (w/v) as a substrate.

### Detection and quantification of ingested esculin

For the detection and quantification of esculin, male and female sand flies were collected after emergence and kept in rearing cages as described above. Insects were offered an esculin-supplemented sucrose solution (70% sucrose, 0.1% esculin w/v) in cotton wool pads *ad libitum* for seven days. Negative control flies were offered non-supplemented sucrose solution. After seven days, whole insects were individually homogenized in 100 μl of solvent (water or methanol) or photographed on a camera under UV radiation. Forty microliters of this homogenate was transferred to a fresh tube, and 200 μl of sodium carbonate buffer (pH 10) was added. Samples were mixed, and fluorescence was read at Ex/Em = 366/432 nm. Quantification of fluorescence was performed using water and methanol as a solvent. Stability of fluorescence in methanol was evaluated by collecting esculin-fed insects in this solvent and then measuring fluorescence after 1, 14 and 28 days. Tissue-specific quantification of esculin by fluorescence was performed as above. However, instead of whole flies, flies were dissected after seven days of esculin-supplemented feeding. Individual crops, midguts and the rest of the bodies were collected and kept in methanol for one day before homogenization and fluorescence reading. The amount of ingested esculin (amount of sugar solution) was calculated from a standard curve of the compound in the corresponding solvent (water or methanol).

### Detection and quantification of ingested mandelonitrile

Fifty flies were placed in cages, as previously described and fed on 70% sucrose containing red dye (Arcolor, São Paulo, Brazil) at a concentration of 50% (v/v) containing 0.1% (w/v) mandelonitrile, or only sucrose with the colorant (control). After two days of feeding, these insects were dissected and crops, midguts and the rest of the bodies were separated. Crops, midguts and the rest of the bodies were homogenized individually in 3, 5 and 20 μl of deionized water (MilliQ, Millipore, Burlington, EUA), respectively. Midgut and the rest of body samples were centrifuged at 12000× *g* for 8 min at 4 °C for the separation of the soluble portion from precipitated tissue fractions. For quantification, absorbance values of 510 nm (maximum wavelength for red dye) of 1 μl of the sample were measured on a Nanodrop 2000C (Thermo, Walthan, USA) with the UV-visible option. Calculations of the amount of ingested dye (and a corresponding amount of sucrose solution) were performed with a standard curve of the dye diluted in water and measured in the same equipment.

### Mandelonitrile attractiveness/repellence assays

Twenty sand flies (male or female) were placed in two acrylic cages. Each cage measured 25.5 cm in height, 25.5 cm in width and 70 cm in length and had an internal wall with holes for the passage of insects, separating the cage in two compartments measuring 24 and 46 cm in length, respectively. The larger side contained a 0.2 g piece of cotton soaked in 2 ml of a 70% (w/v) sucrose solution (control) or sucrose with 0.1% (w/v) mandelonitrile. The sand flies were inserted on the smaller side, and after 1, 2, 3 and 4 h, the number of insects migrating to the other side was recorded.

### *In vitro* effects of glycosides and aglycones in *Leishmania* (*Viannia*) *amazonensis*

*Leishmania* (*Viannia*) *amazonensis* was supplied by Dr Eduardo Caio Torres dos Santos (IOC-FIOCRUZ). Promastigotes (final concentration 1 × 10^6^ parasites/ml) were transferred to Schneider’s medium (24 g/l) containing 10 mg/ml gentamicin, 10% (v/v) inactivated fetal bovine serum, 2% (v/v) human urine and the respective test compound (amygdalin, esculin or mandelonitrile).

Solutions of each test compound [0.1% (w/v)] in phosphate-buffered saline (PBS) were prepared and added 1:9 to the culture medium. The mixture was sterilized by filtering through a 0.22 μm porous filter. Final concentrations were 0.22 mM amygdalin, 0.26 mM esculin, and 0.75 mM mandelonitrile. For controls, the medium was mixed with 1 ml of sterile PBS in the same proportion. After 24, 48 and 72 h at 26 ºC the parasites were observed and counted in a Neubauer chamber using 0.4% (w/v) trypan blue and 2% (v/v) formaldehyde in PBS [35, 36].

### *In vitro* effects of glycosides and aglycones in different species of *Leishmania* spp.

All strains were obtained from the *Leishmania* Collection of Instituto Oswaldo Cruz (CLIOC). Strains used were *L.* (*L.*) *amazonensis* IOC/L0575 (IFLA/BR/1967/PH8), *L.* (*V.*) *braziliensis* IOC/L0566 (MHOM/BR/1975/M2903), *L.* (*L.*) *infantum* IOC/L2906 (MHOM/BR/2002/LPC-RPV) and *L.* (*L.*) *mexicana* IOC/L0561 (MHOM/BZ/1982/BEL21). The experiment was carried out according to the anterior item. The initial concentration of parasites was 5 × 10^5^ promastigotes/ml, and the parasite counting was performed only after 72 h of incubation at 26 ºC with test compounds (or controls). In this assay, the effect of esculetin (final concentration of 0.56 mM) was also observed. Stock solutions of esculetin 1% (w/v) were prepared in NaOH 10% and added 1:9 to PBS before addition in a ratio of 1:9 to a culture medium. For controls, the medium was mixed with 1 ml of sterile PBS or 1 ml PBS with NaOH in the same proportion.

### Dose dependence of the *in vitro* effect of mandelonitrile on *Leishmania* spp. development

The experiment was performed as described above [sections “*In vitro* effects of glycosides and aglycones in *Leishmania* (*Viannia*) *amazonensis*”and “¨*In vitro* effects of glycosides and aglycones in different species of *Leishmania* spp.”], using as final concentrations of mandelonitrile 750 μM, 250 μM, 75 μM, 25 μM and 7.5 μM. PBS was used as a control. Mandelonitrile was added to cultures of *L. amazonensis*, *L. braziliensis*, *L. infantum* and *L. mexicana* (strains as above) initially containing 5 × 10^5^ promastigotes/ml. The results were obtained by counting the number of parasites after 72 h of exposure to the compound in culture at 26 ºC.

### *In vivo* effects of mandelonitrile in *L. longipalpis* infection with *L. mexicana*

Cages were assembled with about 120 adult females of *L. longipalpis*, 3 to 4 days old after emergence from the pupa. The sugary feed was provided for 1.5 days [70% (w/v) sucrose or 0.1% (w/v) mandelonitrile in sucrose 70%]. The cages were kept inside the incubator (BOD) with a temperature of 24 ± 1 °C and humidity in the range of 50–70%.

For infection, citrated rabbit blood was used (with heat-deactivated serum at 56 °C for 1 h in a water bath), containing 2 × 10^6^ parasites/ml of *Leishmania mexicana* promastigote forms in log growth phase (the third or fourth day after the last pass). This protocol was established according to culture observations and growth curve data elaborated for all strains used (data not shown).

The infection was carried out in a Hemotek artificial feeding system (Discovery Workshops, Blackburn, UK). Chick skins were kept at -20 °C until use as a membrane, being previously washed, once with 70% (v/v) ethanol and three times with distilled water. Feeders were connected to the heating system with the temperature stabilized at 37 °C and placed on the cage for females to feed for 1 h.

Two hours after blood-feeding the more engorged females were separated in another cage and the sugars [sucrose 70% (w/v) or mandelonitrile 0.1% (w/v) in sucrose 70%] were again offered *ad libitum*, being exchanged every two days. The cages remained in the BOD with controlled temperature and humidity until the 7th day of infection, when the guts were dissected. Each gut was homogenized manually in 20 μl PBS, 10 μl of which was separated for counting in a Neubauer chamber by adding 5 μl of 0.4% (w/v) trypan blue in PBS and 5 μl of 2% (v/v) formaldehyde in PBS. The remaining 10 μl were used for parasite quantification by qPCR (see below). This protocol was adapted from the procedure already described [37].

### qPCR measurements of *L. mexicana* in infected *L. longipalpis*

#### Sample preparation for a standard qPCR curve

A sample of *L. mexicana* in culture was centrifuged for 10 min at 770× *g* for media separation. A new suspension was prepared in 0.5 ml sterile-filtered PBS at a final concentration of 6.6 × 10^7^ parasites/ml. Previously, 8 gut samples were prepared from *L. longipalpis* adult females in 1.5 ml microtubes. Insects were 3–4 days old after emergence and fed on 70% (w/v) sucrose solution. The midguts were homogenized in 20 μl sterile-filtered PBS and had their volume adjusted to 90 μl with PBS. Ten microliters of the parasite suspension was added to the first tube and mixed. From this mixture, 10 μl was withdrawn, and the procedure was repeated for obtaining serial dilutions in the other seven tubes, resulting in 8 different parasite concentrations, ranging from 2 × 10^6^ to 2 × 10^-1^ parasites/gut.

#### Preparation of the experimental samples

As reported above, each gut from an infected sand fly was homogenized in 20 μl PBS, and 10 μl was withdrawn and frozen at -20 °C until use. Both samples from infected *L. longipalpis* fed on sucrose 70% (w/v) (control) and fed on sucrose 70% (w/v) plus 0.1% (w/v) mandelonitrile (experimental) were analysed.

#### Sample lysis

Gut samples were homogenized in 100 μl 1× TE buffer (10 mM Tris-HCl pH 9.2, 10 mM EDTA) and incubated for 2 h at 56 °C with 100 μg/ml proteinase K (Invitrogen Life Technologies, Carlsbad, CA, USA) with periodic shaking. After centrifugation at 13,000× *g* for 15 min at 4 °C, the recovered supernatant was heated at 95° C for 15 min for proteinase K inactivation. Samples corresponding to the lysates of cells were stored at -20 °C until total DNA extraction.

#### DNA extraction

One-hundred microliters of each sample lysate was subjected to DNA extraction using the commercial Wizard SV Genomic DNA Purification System kit (Promega, Madison, WI, USA) according to the specifications of the manufacturer. The recovered DNA was eluted in 100 μl TE 0.1× and stored at -20 °C until use.

#### qPCR reactions

A real-time qPCR system based on the fluorescent dye SYBR green (Applied Biosystems, Foster City, CA, USA) was used for this study. Two pairs of primers were used. The first pair amplifies the constant region of *Leishmania* minicircle kDNA: primer A (5'-(G/C)(G/C)(C/G) CC(A/C) CTA T(A/T)T TAC ACC AAC CCC-3') and primer B (5'-GGG GTA TTC GGG GCG TGC GAA-3') [38]. The second pair of primers amplifies a gene specific to sand flies (RP49), F (5'-GAC CGA TAT GCC AAG CTA AAG CA-3') and R (5'-GGG GAG CAT GTG GCG TGT CTT-3') [39]. The pair of primers specific for sand flies was needed as a control for the parasite quantification assays, as well as to evaluate the homogeneity of the extraction of DNA from tissue lysates. This procedure allowed the correct quantification of the positive pools. Reaction mixtures consisted of 7.5 μl SYBR green, 2 μl of each primer (forward and reverse, 0.6 pmol/μl), 1.5 μl of molecular biology grade water and 2 μl (150 ng) of DNA, with a total volume of 15 μl. Reactions were incubated at 95 °C for 10 min (activation), then 36 cycles of 95 °C for 30 s (dissociation), 55 °C for 30 s (annealing), 72 °C for 30 s (amplification) and a final stage of 72 °C for 5 min [40]. qPCR reactions were performed on a 7500 FAST equipment (Applied Biosystems) at the IOC-FIOCRUZ real-time PCR facility. The same system was used for data collection and analysis.

Standard curves with predetermined numbers of promastigotes or DNA concentrations were replicated in each assay for calibration. All assays were performed with intra-replica (triplicates of samples), and inter-experiments (at least, three repetitions) replicates.

### Statistical analyses

All data were analyzed using GraphPad Prism version 6 software for Windows. The longevity curves were analyzed by the survival analysis test included in this software. Gaussian normality of data distributions was observed by the D’Agostinho & Pearson test. Samples showing a normal distribution were submitted to Student’s t-parametric tests (non-paired), and samples deviating from normal distribution were compared by the non-parametric Mann-Whitney test.

The results of the parasite count in cultures were submitted to an analysis of variance (one-way ANOVA ), multiple comparisons tests, and confirmed by paired t-tests. Percentages of infected insects were compared using Fisher’s exact test. All results with a *P*-value < 0.05 were considered as statistically significant.

For qPCR parasite load quantitation in infected sand flies, outlier tests were conducted using the C_t_ values of the endogenous control (RP49 gene). No outliers were detected, so all measurements were used for the comparison of groups.

## Results

### Longevity of adult *L. longipalpis* fed on supplemented sugar meals

To assess the effects of some β-glycosides and aglycones in the longevity of adult *L. longipalpis* after emergence from pupae, these compounds were added to sugar meals.

**Fig. 2:**
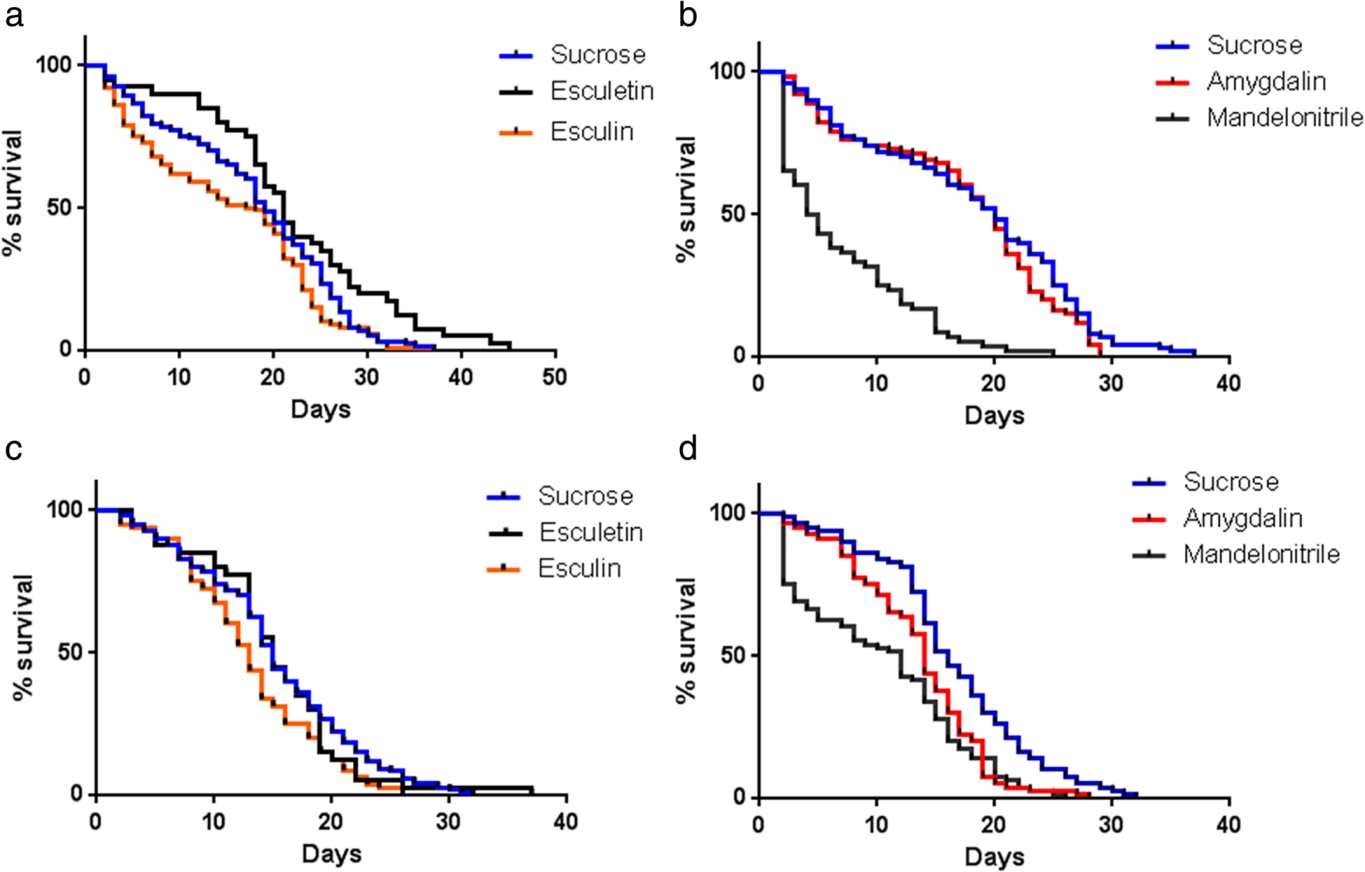
Longevity curves of adult *L. longipalpis* maintained on diets supplemented with different β-glycosides/aglycones. **a** Males were fed on esculin and esculetin *P* = 0.001 (log rank-Mantel Cox); **b** Males were fed on amygdalin and mandelonitrile *P* < 0.0001 (log rank-Mantel Cox); **c** Females were fed on esculin and esculetin *P* = 0.02 (log rank-Mantel Cox); **d** Females were fed on amygdalin and mandelonitrile *P* < 0.0001 (log rank-Mantel Cox). Control flies were fed on a non-supplemented sucrose solution. Significance was considered when *P* < 0.0001 (log rank-Mantel Cox). Data for male survival curves was obtained from 2 to 5 biological replicates using 20 insects in each cage: sucrose (*n* = 100), amygdalin (*n* = 100), esculin (*n* = 100), mandelonitrile (*n* = 60) and esculetin (*n* = 40). Data for females’ survival was obtained from 2 to 4 biological replicates, using 20 insects in each cage: sucrose (*n* = 80), amygdalin (*n* = 80), esculin (*n* = 80), mandelonitrile (*n* = 80) and esculetin (*n* = 40). All insects were maintained in a BOD-type incubator under controlled temperature (26 ± 1°C) and humidity (53–70%)

Figure 2 shows male and female longevity curves and the effect of the addition of test compounds to the sugar meals. Some compounds resulted in significant changes (*P* < 0.0001) in the mean survival values. The average lifespan (ALS) of control males, fed on sucrose only, was 17 days. Feeding males on esculin reduced the ALS to 15 days (12% reduction), while esculetin seemed to increase their longevity (29% increase, *P* < 0.02). Male ALS was not affected by amygdalin, and mandelonitrile reduced ALS to 7 days (reduction of 59%). Females fed on sugar only showed an ALS of 15 days (Fig. 2). Esculin decreased the ALS by one day (reduction of 13%), and esculetin-fed females showed no variation in ALS. Amygdalin decreased the ALS by one day (reduction of 13%), while mandelonitrile reduced the ALS to 10 days (reduction of 33%). Table 1 summarizes these results.

**Table 1:** Lifespans of adult *Lutzomyia longipalpis* reared on different β-glycosides/aglycones-supplemented diets. Insects were separated and followed after emergence from pupae

		Sucrose	Amygdalin	Esculin	Mandelonitrile	Esculetin
Males	Median (days)	19	20	18	5	21^b^
	Mean (days) ± SEM	17 ± 1	17 ± 1	15 ± 1	7 ± 1^a^	22 ± 1^b^
	Mean reduction (%)		0	12	59	-29
Females	Median	15	14	13	12	15
	Mean ± SEM	15 ± 1	13 ± 1^c^	13 ± 1^d^	10 ± 1^e^	15 ± 1
	Mean reduction (%)		13	13	33	0

Values are expressed in days ± standard error of the mean (SEM). An asterisk indicates statistical difference (Paired t-test), comparing control and experimental groups ^a^*P* < 0.0001 (two-tailed), *t* = 5.651 *df* = 59; ^b^*P* < 0.0001, *t *= 4.378 *df* = 39; ^c^*P* < 0.0001, *t* = 5.390 *df* = 79; ^d^*P* < 0.0001, *t* = 12.47, *df* = 79; and ^e^*P* < 0.0001, *t* = 8.922, *df* = 79

### Effects of esculin and esculetin on different *L. longipalpis* carbohydrases

#### Esculin hydrolysis

After recording the effects of esculin on sand fly longevity, we decided to study if this compound has some effect on its known targets such as trehalase and β-glucosidase described in other insects [24]. Because the effect of esculin on ALS was minimal, we expected that those enzymes might be refractory to this compound. However, before testing this hypothesis, we decided to verify if esculin is hydrolyzed by some endogenous enzyme. Tissue homogenates from both females and males, fed on sugar only, presented significant esculin hydrolysis activity with no significant difference between sexes (Additional file 1: Table S1). Interestingly, we found similar amounts of esculin hydrolysis activity in midgut and rest of body, with higher activities in the gut contents and in the soluble fraction of the rest of body (Additional file 1: Table S1).

After realizing that esculin might be hydrolyzed at significant rates in sand fly tissues, we decided to look if diets containing esculin would change these endogenous activities, to avoid the release of the toxic aglycone esculetin or increase its detoxification rate. Esculin-fed males showed somewhat lower activities in the gut contents and the rest of body tissues (Additional file 1: Table S2), and females showed lower activity in the rest of body tissues (Additional file 1: Table S3). Nevertheless, due to the high variability of the data, these differences were not statistically significant.

#### Trehalase activities

Since trehalase is the target enzyme of several toxic plant glycosides in other insect species, we investigated the effects of esculin on sand fly trehalase activity. In these tests, only values obtained in the presence of proteolysis inhibitors were reliable, since sand fly trehalase was not stable in their absence (data not shown).

We detected a substantial trehalase activity in all tissues investigated, in both females and males (Table 2 and Additional file 1: Table S4). There were no significant changes in trehalase activity in male samples when the assays are performed in the presence of esculin (Additional file 1: Table S4). Contrastingly, female samples displayed *in vitro* inhibition of trehalase activity in the tissues and soluble fraction of the rest of the body (Table 2, *P* < 0.05).

**Table 2:** *In vitro* effects of esculin on trehalase activity of adult female *Lutzomyia longipalpis*. Tissue homogenates were incubated or not with esculin, and enzyme assays were performed using trehalose as a substrate. Tissues were homogenized and separated by centrifugation into gut tissue, gut contents, rest of body tissues and rest of body soluble fraction. Results are presented as μU/insect and are the mean and SEM of 8 biological replicates with 4 insects in each sample

Sample	Control (trehalose only)	Esculin added to the assay mixture
Gut, tissue	380 ± 70	300 ± 100
Gut, contents	170 ± 60	220 ± 90
Rest of body, tissues	560 ± 60^a^	380 ± 50^a^
Rest of body, soluble fraction	580 ± 10^b^	360 ± 20^b^

^a^Mann-Whitney test, *U* = 13, *P* = 0.046

^b^Mann-Whitney test, *U* = 0, *P* = 0.0006

We also studied if feeding sand flies on an esculin-supplemented sucrose diet would change the trehalase activities in different tissue homogenates. No significant variations in enzymatic activity were registered in esculin-fed male (Additional file 1: Table S5) or female flies (Additional file 1: Table S6) when compared to controls.

To have a better understanding of the physiological effects of esculetin-supplemented diets on sand fly metabolism, we decided to study the *in vitro* effects of esculetin (the aglycone of esculin) on *L. longipalpis* adult trehalase activities. For males, we observed a tendency for inhibition in gut and rest of body tissues, and rest of body soluble fraction, although without significance (Table 3). Besides that, the activity of the rest of the body tissues of females was significantly inhibited (Table 4). It is important to mention that for doing these measurements the usual assay procedure had to be adapted because esculetin interferes with the tris-glucose-oxidase kit (TGO) used to reveal the released glucose from trehalose. This was solved by treating the aliquots after interruption of each time-point with potassium dichromate, before the addition of the TGO reagent (data not shown, see Methods for details).

**Table 3:** *In vitro* effects of esculetin (aglycone of esculin) on trehalase activities of male *Lutzomyia longipalpis* adults. Insects were fed on sucrose only, and esculetin was added in the assay mixtures of different tissues with trehalose as a substrate. Tissues were homogenized and separated by centrifugation into gut tissue, gut contents, rest of body tissues and rest of body soluble fraction. Results are presented as μU/insect and are the mean and SEM of 4 biological replicates with 4 insects in each sample

Sample	Control (trehalose only)	Esculetin added to the assay mixture
Gut, tissue	309 ± 54	208 ± 42
Gut, contents	87 ± 30	120 ± 52
Rest of body, tissues	215 ± 48	96 ± 9
Rest of body, soluble	282 ± 41	177 ± 66

**Table 4:** *In vitro* effects of esculetin (aglycone of esculin) on trehalase activities of female *Lutzomyia longipalpis* adults. Insects were fed on sucrose only, and esculetin was added in the assay mixtures of different tissues with trehalose as substrate. Tissues were homogenized and separated by centrifugation into gut tissue, gut contents, rest of body tissues and rest of body soluble fraction. Results are presented as μU/insect and are the mean and SEM of 6 biological replicates with 4 insects in each sample

Sample	Control (trehalose only)	Esculetin added to the assay mixture
Gut, tissue	290 ± 30	180 ± 60
Gut, contents	190 ± 50	110 ± 30
Rest of body, tissues	400 ± 30^a^	160 ± 40^a^
Rest of body, soluble	300 ± 30	330 ± 50

^a^Mann-Whitney test, *U* = 4, *P* = 0.026

#### β-glucosidase activities

Because esculin and esculetin are known inhibitors or alternative substrates for β-glucosidases of other insects, we decided to investigate if *L. longipalpis* β-glucosidases are affected by these compounds. For these experiments, endogenous activities of esculin hydrolysis were discounted from the corresponding controls with no substrate. Data in Additional file 1: Tables S7 and S8 show that no direct inhibition of cellobiose hydrolyzing activities was observed when adding esculin to male or female adult samples. Some activities as in the rest of the body soluble fraction of females (Additional file 1: Table S8) seem to be diminished, but with no statistical significance.

We also investigated whether the ingestion of esculin would cause changes in β-glucosidase activity in different tissues of adult sand flies. Male tissue homogenates did not show significant variations in enzymatic activities (Additional file 1: Table S9). However, female samples showed a statistically significant increase of activity in the soluble portion of the rest of the body (Table 5).

**Table 5:** Effect of supplementing the sucrose diet with esculin on β-glucosidase activities in female *Lutzomyia longipalpis*. Tissue homogenates were assayed using cellobiose as a substrate. Tissues were homogenized and separated by centrifugation into gut tissue, gut contents, rest of body tissues and rest of body soluble fraction. Results are presented as μU/insect and are the mean and SEM of 5 biological replicates with 10 insects in each sample

Sample	Control (sucrose only)	Esculin supplemented
Gut, tissue	1.3 ± 0.8	2.2 ± 0.8
Gut, contents	8 ± 3	2 ± 1
Rest of body, tissues	1.6 ± 1.0	3 ± 1
Rest of body, soluble	0^a^	8 ± 1^a^

^a^Mann-Whitney test, *U* = 0, *P* = 0.0159

To have a better understanding of the effects of esculin in the activity of β-glucosidase of sand flies, we also tested the *in vitro* inhibition of this enzyme by esculetin, the aglycone part of this glycoside. Esculetin activated the activity from the soluble fraction of the rest of the body from males (Table 6) but had no significant effect on the activity from female samples (Additional file 1: Table S10).

**Table 6:** *In vitro* effects of esculetin on the β-glucosidase activities of male *Lutzomyia longipalpis*. Tissue homogenates were incubated with esculetin and then assayed using cellobiose as a substrate. Tissues were homogenized and separated by centrifugation into gut tissue, gut contents, rest of body tissues and rest of body soluble fraction. Results are presented as μU/insect and are the mean and SEM of 4 biological replicates with 20 insects in each sample

Sample	Control (cellobiose only)	Esculetin added to the assay mixture
Gut, tissue	7 ± 3	0
Gut, contents	14 ± 3	11 ± 4
Rest of body, tissues	4 ± 1	4± 3
Rest of body, soluble	6 ± 2^a^	20 ± 10^a^

^a^Mann-Whitney test, *U* = 0, *P* = 0.0286

### Quantification of the ingested amounts of esculin by *Lutzomyia longipalpis* using fluorescence

**Fig. 3:**
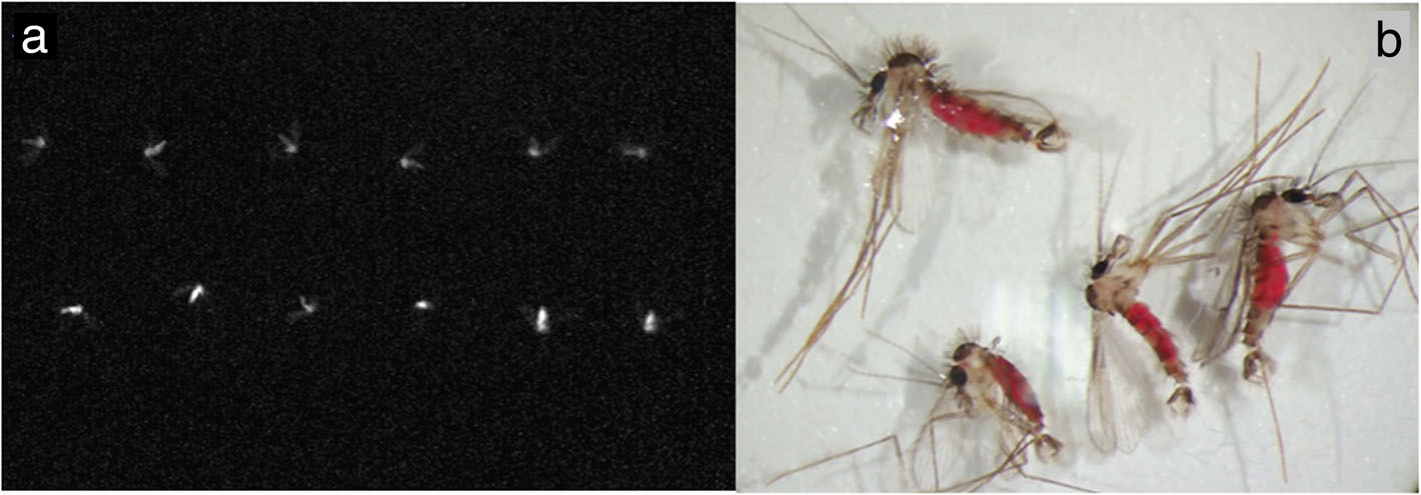
Sugar solution ingestion by *Lutzomyia longipalpis* adults fed on sucrose diets containing esculin or mandelonitrile. **a** The image was taken using a UV-light photographic system (E-gel Imager, Life Technologies). Top row: sucrose-fed females; bottom row: sucrose + esculin-fed females. **b** Males of *L. longipalpis* after two days of feeding *ad libitum* on sucrose diets with red food colorant and mandelonitrile (magnification 25×)

Esculin fluoresces under longwave ultraviolet (UV) light. To confirm that adults fed on this compound, sand flies were maintained on esculin-supplemented sucrose and photographed under UV-radiation. Esculin-fed males and female *L. longipalpis* showed fluorescence in the whole body, unlike insects fed on sucrose only (Fig. 3a).

To measure the amount of ingested esculin, the insects had their whole body homogenized, and aliquots were taken for fluorescence quantification. Initially, this procedure was carried out using milliQ water as a solvent. However, we decided to use methanol instead to achieve easier immersion of the sand fly body, given the hydrophobic nature of the cuticle. Additionally, methanol as a solvent would be better for the long-term preservation of field samples at room temperature. The stability of ingested esculin in each solvent was evaluated by measuring fluorescence at different time-points after immersion of the sand fly. There were no significant differences between the amounts of ingested esculin between males and females. Extraction with methanol gave similar results after 14 days of immersion, and the fluorescence was stable until 28 days of incubation at room temperature. However, incubation of male sand flies in methanol for only one day before homogenization resulted in lower fluorescence values (Table 7).

**Table 7:** Quantification of the ingested esculin by *Lutzomyia longipalpis* adults exposed to esculin-supplemented diets. Flies were maintained on an esculin-supplemented sucrose diet for 6 to 7 days. Insects were immersed and homogenized in two different solvents, water or methanol. In the solvent test, flies were immersed for 8 days. Stability of fluorescence was evaluated by immersing esculin-fed insects in methanol and then homogenizing for fluorescence measurements after 1, 14 or 28 days. Basal fluorescence of the negative controls (sucrose-fed only) was subtracted from experimental sample values. Each sample used for extraction and measurement consisted of an individual sand fly. Results are shown in pmol/insect and are the mean and SEM of 3 biological replicates, with a total measurement of *n* = 22 (male) and *n* = 24 (female) in solvent tests and *n* = 29 (male) and *n* = 36 (female) in stability tests

Solvent	Incubation time in the solvent (days)	Males (pmol/insect)	Females (pmol/insect)
Water	8	58 ± 8	67 ± 9
Methanol	8	52 ± 7	70 ± 7
Methanol	1	34 ± 4^a^	53 ± 5
Methanol	14	49 ± 5	47 ± 6
Methanol	28	49 ± 5	52 ± 7

^a^Mann-Whitney test, *U* = 106.5 *P* = 0.01

To confirm that esculin was actually ingested and not just adhered on the surface of the insect cuticle, we repeated the previous experiment but this time midgut, crop and rest of body were dissected and assayed separately. Table 8 shows tissue-specific amounts of ingested esculin for males and females, as well as the values for entire sand flies obtained in similar conditions (from Table 7). The sum of the esculin-ingested amounts of the midgut, crop and carcass was 39 pmol/insect in males and 56 pmol/insect in females. These values were very close to those obtained in the previous experiments, 34 and 53 pmol/insect in males and females, respectively (Table 7). Esculin-ingested amounts were statistically different between males and females in different tissues (ANOVA, *P* < 0.0001). Samples from female crop and carcass displayed higher amounts when compared with those in males (*P* < 0.05), but midgut values were not different between the two groups (Table 8).

**Table 8:** Quantification of the esculin amount in different tissues of *Lutzomyia longipalpis* after exposure to esculin-supplemented diets. Insect adults were fed *ad libitum* on sucrose plus esculin for 7 days. After that, the flies were immersed individually in methanol and tissues were dissected and homogenized on the same day of collection from the cage. Basal fluorescence of the negative controls (sucrose-fed only) was subtracted from experimental sample values. Results are shown in pmol/insect and are the mean and SEM of 3 biological replicates with a total of *n* = 36 (male) and *n* = 29 (female)

Sample	Males (pmol/insect)	Females (pmol/insect)
Whole body	34 ± 4	53 ± 5
Crop	28 ± 1^a^	43 ± 6^d^
Midgut	7 ± 1^b^	6 ± 1^b^
Rest of body	4 ± 1^c^	7 ± 2^b^

Equal superscript letters indicate groups with no significant statistical difference between them (Mann-Whitney tests, *P* < 0.05)

Based on the fluorescence measurements above, we estimated the total volume of esculin + sugar solution and the total amount of sugar ingested per insect. Table 9 illustrates the values obtained after conversion of the data in Table 8.

**Table 9:** Volumes of the esculin + sugar solution and the total amount of sugar ingested by *Lutzomyia longipalpis* adults. Insects were fed for 7 days *ad libitum* on esculin-supplemented sucrose. Results are shown in ηl/insect or μg/insect and are the mean and SEM of 29 (male) and 36 (female) individual samples

Ingested amount	Females	Males
Volume of solution (ηl)	20.4 ± 1.9	13.1 ± 1.5
Total sugar (μg)	14.3 ± 1.3	9.1 ± 1.1

### Colorimetric quantification of ingested mandelonitrile

Insects were offered sugar meals containing red food colorant, supplemented or not with mandelonitrile, to confirm its ingestion. The amount of red dye ingested was used to calculate the amount of sugar solution ingested *via* colorimetric measurement. Only the crop showed significant absorbance values, as midgut and carcass samples showed absorbances similar to controls offered water only, with no dye added (data not shown). Table 10 provides the estimated volume of ingested solution by flies offered sugar or sugar plus mandelonitrile, using the crop data only. No significant differences were found between the sexes or between the different diets. Figure 3b shows that the insects from both groups are visually very similar, displaying the same red coloration.

**Table 10:** Volumes of sugar solution ingested by *Lutzomyia longipalpis* after 2 days of exposition to sugar diets containing red dye. Insects were fed on sucrose only (control) or sucrose plus mandelonitrile. The amounts of sugar solution ingested were obtained from a standard curve performed with the red dye and subsequent calculations. The values are mean and SEM in ηl/insect obtained from 18 to 21 samples (individual measurements)

	Males, sugar	Males, sugar + mandelonitrile	Females, sugar	Females, sugar + mandelonitrile
Volume of solution (ηl)	23 ± 2	19 ± 3	22 ± 3	26 ± 3

### Mandelonitrile attractiveness/repellence assays

**Fig. 4:**
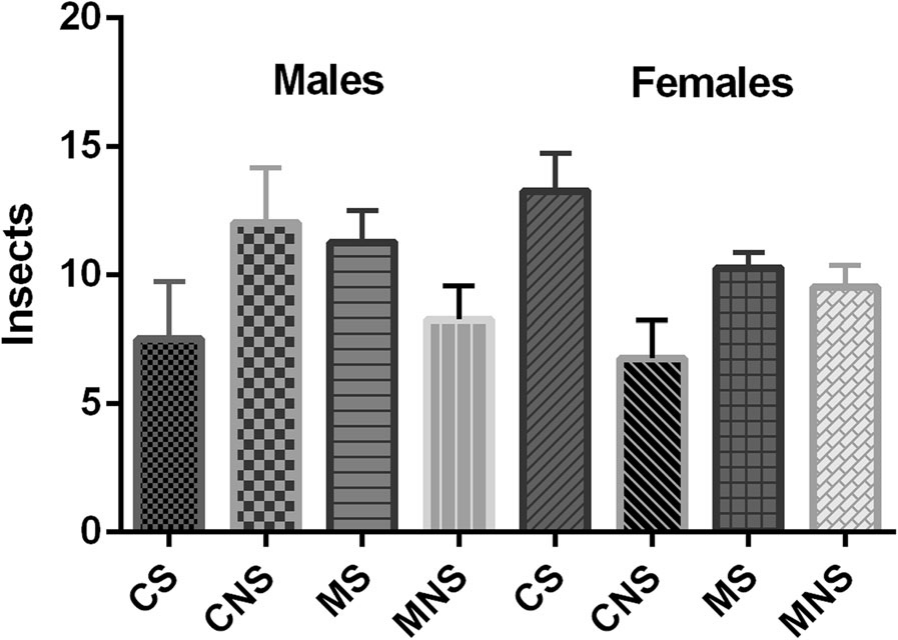
Mandelonitrile attraction/repellence in male and female adults of *Lutzomyia longipalpis*. The number of flies on both sides of the cage was registered 1, 2, 3 and 4 h after the release of flies. The results only after 4 h of experiment are shown, as there were no significant differences along time. No significant difference between groups was observed (t-test). Results are presented are the mean ± SD of four biological replicates with 20 insects each. *Abbreviations*: CS: control experiment, sugar side (big chamber); CNS: control experiment, no sugar side (small chamber); MS: mandelonitrile experiment, sugar side (big chamber); MNS: mandelonitrile experiment, no sugar side (small chamber)

To assess if mandelonitrile has any repellent or attractive effect on sand flies, we carried out attractiveness tests in acrylic cages. There were no significant behavioral differences between experimental and control groups in either male or female sand flies (Fig. 4).

### *In vitro* effects of glycosides and aglycones on different species of *Leishmania*

**Fig. 5:**
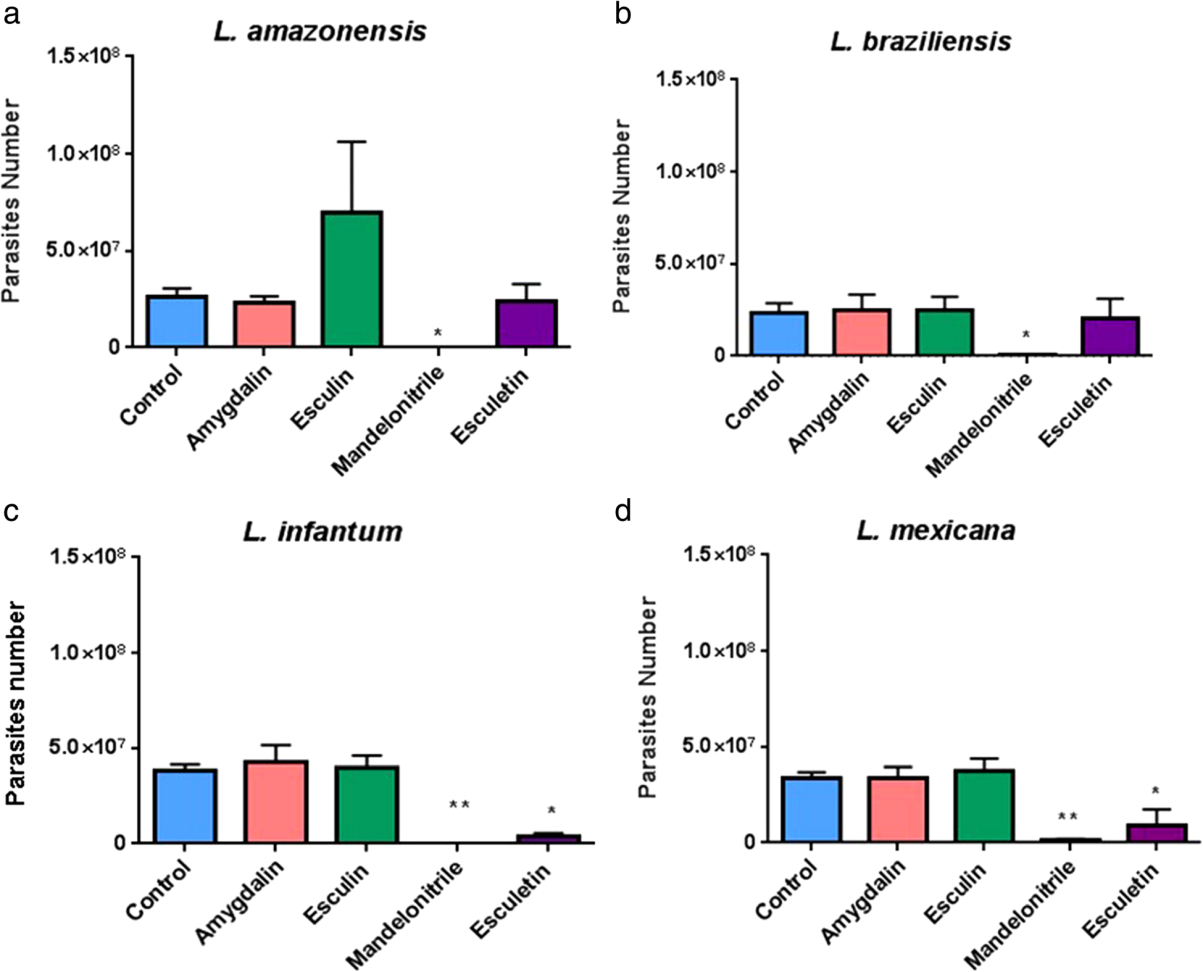
*In vitro* effects of glycosides and aglycones on cultures of different *Leishmania* species. Parasite numbers were measured 72 h after the addition of test compounds (other conditions as in Fig. 4). Glycosides and aglycones were tested on *Leishmania amazonensis* (**a**), *L. braziliensis* (**b**), *L. infantum* (**c**) and *L. mexicana *(**d**). Results shown are the mean and SEM of 4 biological replicates. *P < 0.05, **P < 0.01 (Dunnett’s multiple comparisons test)

To initially analyze the effects of glycosides and aglycones on parasites of different species of *Leishmania*, we incubated a culture of *Leishmania* (*Viannia*) *amazonensis* with 0.01% amygdalin, esculin or mandelonitrile. Data presented in Table 11 show that esculin and amygdalin had no significant effect on parasite numbers. However, mandelonitrile caused a significant reduction in the number of parasites after 24 h (one-way ANOVA, multiple comparisons test, *P* < 0.05). After 48 and 72 h, parasite counts were even lower (Table 11). Subsequently, we performed further experiments to verify if the effects of the compounds tested would be equally observed in *L. amazonensis*, *L. braziliensis*, *L. infantum* and *L. mexicana*. We also included esculetin (560 μM) in these new tests. Figure 5 shows that mandelonitrile significantly decreased parasite numbers in all species tested, while esculetin had a similar effect on both *L. infantum* and *L. mexicana*.

**Table 11:** *In vitro* effects of glycosides and aglycones on the growth of *Leishmania amazonensis* in culture. Parasites were counted with a hemocytometer at 24, 48 and 72 h after adding compounds to a culture with an initial concentration of 1 × 10^6^ parasites/ml. Results shown are the mean and SEM of 3 biological replicates. Controls received only PBS

Time of exposure (h)	Control (× 10^6^)	Amygdalin (× 10^6^)	Esculin (× 10^6^)	Mandelonitrile (× 10^6^)
24	6 ± 1	8 ± 2	9 ± 2	1.1 ± 0.5^*^
48	26 ± 3	32 ± 4	30 ± 6	0.2 ± 0.1^**^
72	58 ± 5	74 ± 9	62 ± 3	0.6 ± 0^***^

^*^P < 0.05, ^**^P < 0.001, ^***^P < 0.0001. Ordinary one-way ANOVA; Unpaired t-test for *post-hoc* multiple comparisons

### Effects of different mandelonitrile concentrations on *Leishmania* spp. *in vitro*

**Fig. 6:**
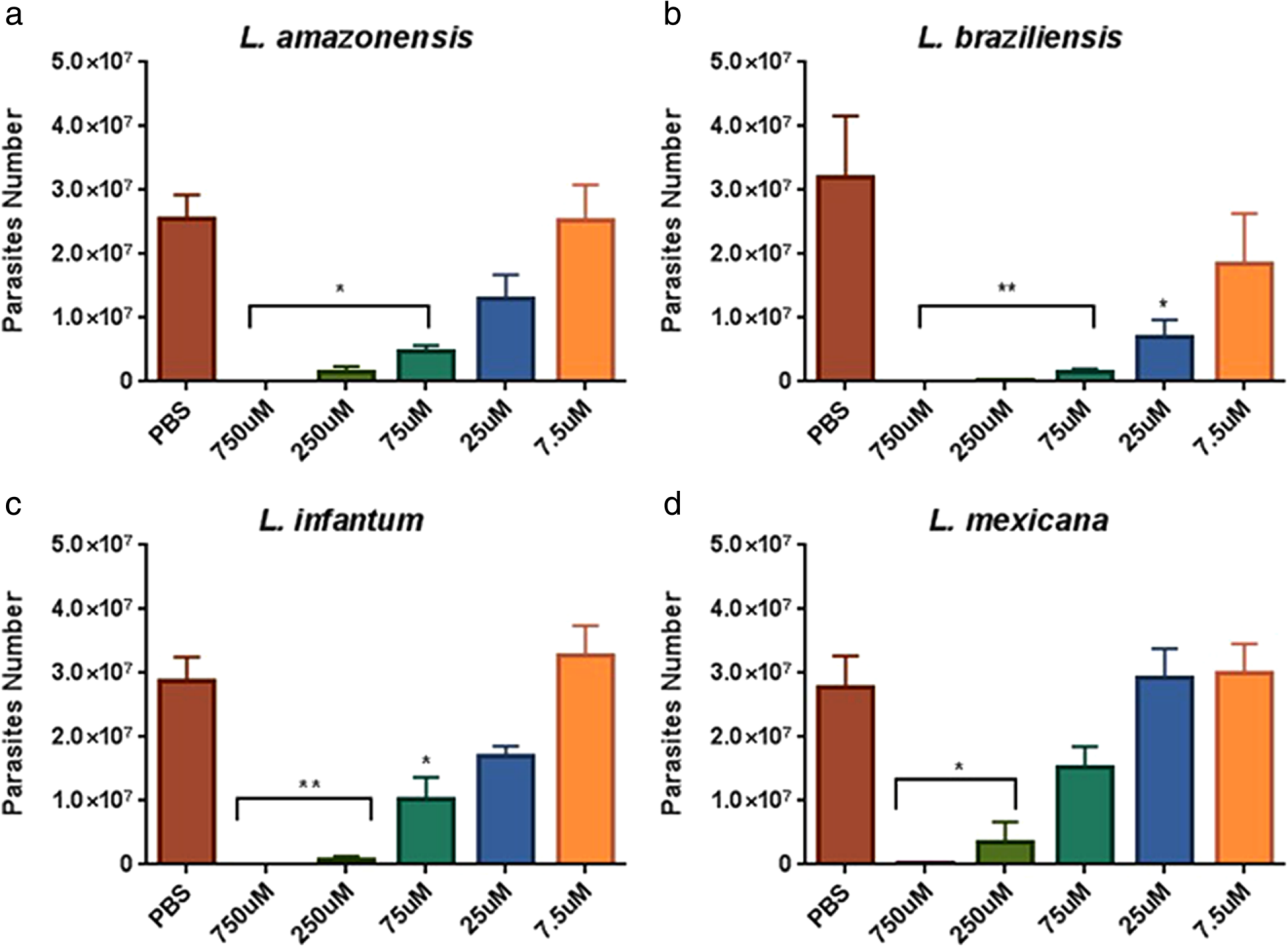
*In vitro* dose-dependent effect of mandelonitrile on *Leishmania* spp. cultures. Parasite numbers were measured after 72 h of incubation in a medium containing an initial concentration of 0.5 × 10^6^ parasites/ml and different concentrations of mandelonitrile. Mandelonitrile was tested on *Leishmania amazonensis* (**a**), *L. braziliensis* (**b**), *L. infantum* (**c**) and *L. mexicana* (**d**). Results shown are the mean and SEM of 3 to 4 independent biological replicates. *P < 0.05, **P < 0.01 (Dunnett’s multiple comparisons test)

Since mandelonitrile exhibited the highest anti-*Leishmania* activity, we decided to study its effects at different concentrations (ranging from 7.5 μM to 750 μM). Mandelonitrile exhibited a dose-dependent effect on all *Leishmania* species tested (Fig. 6). A concentration of 25 μM mandelonitrile is capable of causing a significant adverse effect on *L. braziliensis*, while for the other species, significant reductions were observed within the range of 75–250 μM.

### Mandelonitrile blocking of *L. longipalpis* infection with *L. mexicana*

#### Measurements using Neubauer chamber counts

Based on the *in vitro* results, we decided to verify if artificial infections of *L. longipalpis* females with *L. mexicana* would be affected by rearing the flies on different sugar diets, mandelonitrile-supplemented or not, before and after the blood-feeding. Table 12 presents parasite numbers per female gut and prevalence of infection on the 7th day after blood-feeding on infected blood. We observed that sugar meals containing mandelonitrile 0.1% reduce the number of parasites in the gut of treated sand flies by 65%, as well as the percentage of infected insects from 60 to 40% when compared to flies fed only on sucrose (Fisher’s exact test, *P* = 0.001).

**Table 12:** *In vivo* effect of mandelonitrile supplemented sugar diet on *Leishmania mexicana* infections in female *Lutzomyia longipalpis*. Results are the mean and SEM of hemocytometer counts of female gut homogenates on the 7th day after feeding on *Leishmania*-infected blood. Controls were maintained on sugar only and infected using the same conditions as the experimental group (see Methods for details). Data obtained from 2 biological replicates. Control group *n* = 22, mandelonitrile group *n* = 20

	Control (sugar only)	Mandelonitrile supplemented
Parasites/gut number (mean ± SEM)	10,709 ± 2989	3780 ± 1670
Infection % (females infected/total females × 100)	64	40^a^

^a^Fisher’s exact test, *P* = 0.001

#### Measurements of parasite numbers by qPCR

**Fig. 7:**
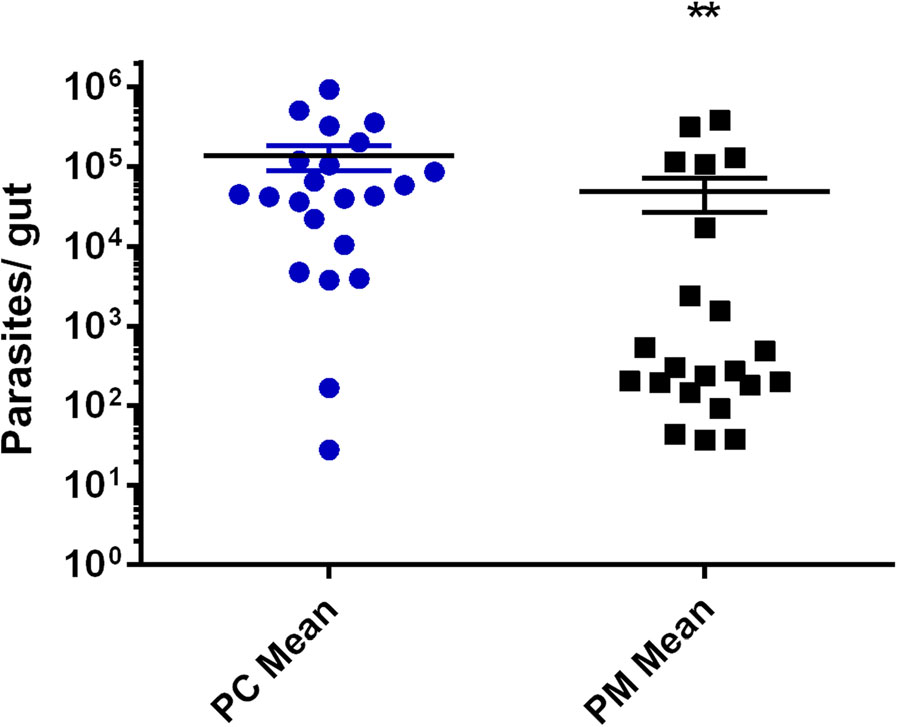
*Leishmania mexicana* parasite numbers in the gut of *L. longipalpis* females on the 7th day after infection (qPCR quantification). The results are presented as parasites per gut, mean ± SEM of 22 samples obtained from 4 independent infection experiments. Mann-Whitney test, *P* = 0.0036. *Abbreviations*: PC, parasite numbers from control flies fed on sugar only; PM, parasite numbers from flies fed on 7.5 mM (0.1% w/v) mandelonitrile-supplemented sugar diets

Differences in parasite numbers after infection of flies fed on sugar only or sugar diets supplemented with mandelonitrile were also assessed using quantitative PCR (qPCR). Female flies reared on mandelonitrile-supplemented sucrose showed significantly lower parasite numbers when compared to control insects (Mann-Whitney test, *P* = 0.009; Fig. 7). Quantifications of all control and experimental samples were within the approximate range of the standard curve (Additional file 2: Figure S1; Additional file 3: Figure S2).

## Discussion

### Effects of esculin and esculetin

The main results obtained in this study are summarized in Table 13. Initially, we showed the differences in longevity between sexes and the changes in the ALS of *L. longipalpis* when exposed to diets containing selected glycosides/aglycones. Males had a longer ALS than females (17 *vs* 15 days), which agrees with the longevity results recorded for *Phlebotomus papatasi*. This species reared on sucrose exhibited an ALS for females of 17 days [41].

**Table 13:** Summary of results by compound

Compound	Longevity reduction (%)		Trehalase activities		β-glucosidase activities		Quantification sugar ingested (ηl)		Anti-*Leishmania*	
	Males	Females	Males	Females	Males	Females	Males	Females	Culture	Infection
Amygdalin	ne	13	nt	nt	nt	nt	nt	nt	ne	nt
Esculin	12	13	ne	RBT/RBS inhibition (*ivt*)	ne	RBS activation (*ivv*)	20.4 ± 1.9	13.1 ± 1.5	ne	nt
Mandelonitrile	59	33	nt	nt	nt	nt	nt	nt	Yes, all species tested	nt
Esculetin	-29	ne	GC inhibition (*ivt*)	RBT inhibition (*ivt)*	ne	RBS activation (*ivt*)	19 ± 3	26 ± 3	Yes, *L. infantum/L. mexicana*	Yes, *L. mexicana*

*Abbreviations: ivt in vitro*, *ivv* *in vivo*, *nt* not tested, *ne* no effect, *GT* gut tissue, *GC* gut contents, *RBT* rest of the body tissues, *RBS* rest of the body soluble fraction

Esculin-induced physiological damage has been already observed in other insects. *S. frugiperda* larvae, when reared in 0.1% (w/v) esculin, showed reduced growth and weight compared to controls. This effect resulted in weight loss in female pupae [27]. Synthetic coumarins also have an insecticidal effect on *D. melanogaster* larvae, in addition to its antibacterial activity. These glycosides are plants heterocyclic compounds with several applications, such as insecticides, antibiotics and analgesics, among others [42].

Esculin and esculetin are coumarins found in plants with well-documented biological properties, including significant anti-cancer activity [43]. The extension of ALS observed in male sand flies treated with esculetin suggests that this compound is not acting only as a harmful alkylating agent in this insect. Otherwise, it seems to be beneficial to the sand fly metabolism, similar to the observed effect in mammals.

On the other hand, the effect of different types of plant allelochemicals on the growth and development of insects has been studied. Umbelliferone (7-hydroxycoumarin) results in a small inhibition of ecdysone-20-monooxygenase activity in the midgut and fat body of *Aedes aegypti*, *Drosophila melanogaster* and *Manduca sexta* [44]. Ecdysone-20-monooxygenase is an insect steroid hydroxylase responsible for the conversion of ecdysone to its active form, 20-hydroxyecdysone [44]. This hormone is mainly involved in the processes of molting and metamorphosis. It is also involved in the production of vitellogenin, behavior, resistance to stress and longevity [45, 46]. Previously studies [44] compared ecdysone-20-monooxygenase with vertebrate-dependent cytochrome P-450 steroid hydroxylase, suggesting ecdysteroids as good targets for the action of plant compounds on insect resistance.

The effects reported above for dipterans might be related to the reduction in longevity observed in esculin-fed *L. longipalpis*. In this respect, it would be necessary to analyze the variations in the activity of ecdysone-20-monooxygenase in treated sand flies. That would be especially relevant for males, which did not present changes after treatment with esculin in any of the several candidate target enzymes studied here (see below).

The similar esculinase activity in both sexes is consistent with the impact of this glycoside in longevity since both presented a similar reduction in ALS when exposed to this compound. Interestingly, we did not observe significant changes in esculinase activity after feeding the insects on esculin. The absence of changes in esculinase activity under these conditions, where ALS is reduced, reinforces the observation that adult sand flies are not adapted to the ingestion of toxic glycosides.

Glucosidase-mediated esculin hydrolysis occurs in several microorganisms. This biochemical process is a property used for the identification of the genus *Streptococcus*, as well as *Enterobacteriaceae* such as *Enterobacter* spp., *Escherichia coli*, *Klebsiella pneumonia* and *Serratia marcescens*, among others [47, 48]. It is possible that some esculinase activities described here are in fact from the gut microbiota. Such fact could explain the high variability observed among samples, as gut microbiota varies between individuals in number and composition. It has been shown [49] that *L. longipalpis* has a rich gut microbiota and that *Enterobacter cloacae* and *Serratia marcescens* are their most abundant species. Further research should explore the putative role of gut bacteria in detoxification of these compounds. Participation of gut microbes in the resistance to toxic glycosides has already been described for *Tenebrio molitor* (Coleoptera) [50] and might be a critical issue for the use of these compounds as a strategy for insect control.

The *in vitro* inhibition of trehalase in female rest of body samples by esculin might explain the reduction in the ALS of females fed on this glycoside. Other studies found similar results. Some insect species modulate the activity of trehalase to overcome the damage caused by inhibiting glycosides [24]. However, esculin-fed sand flies exhibited no changes in endogenous trehalase levels. This observation was somewhat expected since there was a minor reduction in insect longevity, and no *in vitro* inhibition of the enzyme by the compound in most tissues. Since it is a vital enzyme in insect metabolism, trehalase inhibition could lead to elevated mortality rates, as previously described in *S. frugiperda* larvae [27]. Despite the ALS reduction in both sexes, only inhibition in female trehalase was observed *in vitro*. In this way, adult sand fly trehalase may have different properties depending on sex.

The absence of trehalase modulation also suggests that sand flies might not be well adapted to feed on toxic glycosides and this consistent with the fact that toxic glycosides are usually not the most important components of insect diets [27]. However, recent studies in field populations of *L. longipalpis* from Teresina, Brazil [51], showed a dietary preference for plants of the families Fabaceae and Rutaceae, in which several secondary metabolites are present, such as coumarins. Thus, more studies are needed to identify which plant components are present in the diet of wild sand flies.

Interestingly, we observed *in vitro* inhibition in trehalase activity in some tissues with the addition of esculetin, but longevity data did not show any deleterious physiological effect of this compound. Additionally, it has been hypothesized that esculetin might add alkyl radicals in the imidazole group of the active center of insect chymotrypsins, rendering it inactive [27]. However, since these flies were not blood-fed, the deleterious effects due to the inhibition of chymotrypsins might not be detectable.

To our knowledge, this is the first record of trehalase inhibition by esculetin in adult dipterans, which makes it difficult to perform a comparative analysis with other insects. Studies in mammals, however, show several beneficial effects of esculetin, such as the elimination of hydroxyl radicals, protection of bio-molecules against oxidative damage by reactive oxygen species, as well as antioxidant and anti-inflammatory activity [43, 52]. It has been shown that reactive oxygen species have important roles in sand fly physiology [37, 53], so the esculetin mechanism might involve changes in the sand fly overall redox balance.

Esculetin prevents oxidation reactions of other molecules, probably by it being itself oxidized. As such, esculetin could be considered a reducing agent. That was evident from the fact that in our trehalase activity assays in the presence of esculetin, it was necessary to treat the samples with the strong oxidative K_2_Cr_2_O_7_ before the measurement of glucose with the Tris-glucose oxidase reagent. In general, these results showed that even with the putative alkylating and detrimental activity, this aglycone seems to be involved in antioxidant pathways, exhibiting a positive effect on the insect’s metabolism.

We also studied β-glucosidase activities, but there was a very low activity of this enzyme in adult phlebotomines. These data are similar to that described in [54], where the plant-feeding dipteran *Rhynchosciara americana* exhibited the lowest β-glucosidase activity when compared to other predatory species. Besides that, due to the characteristic fluorescence of esculin, it is not possible to estimate its inhibition of β-glucosidase by a direct fluorimetric method with methylumbelliferyl-derived substrates, as the inhibitor would interfere in the detection of the product released. So we were limited to the study of cellobiose hydrolysis with the detection of glucose. In this respect, it was more difficult to obtain reliable data, due to the low absorbance values and high biological variability. The recent development of more sensitive glucosidase assays [55], using single flies, might be a powerful tool to overcome this problem.

The increase of β-glucosidase activity only in the rest of the body soluble fraction of esculin-fed females is consistent with the specific inhibition by this compound of female trehalase. Thus, the physiological response of females to esculin might be through the modulation of β-glucosidase instead of changes in the trehalase activity, although the results of esculin hydrolysis experiments did not support this hypothesis. Alternatively, this β-glycoside might be partially or wholly hydrolyzed by the insect’s microbiota.

The β-glucosidases of the moths *Spodoptera frugiperda* [23] and *Diatraea saccharalis* [56] have also been studied. Differences in substrate specificity in midgut β-glucosidases have also been found in different insect orders [56]. Overall, two or three “A” or “B” type β-glucosidases [24] have been described for substrates with hydrophilic or hydrophobic aglycones, covering the different amygdalin hydrolysis stages for mandelonitrile production. However, there is still no description of the mechanism of esculin hydrolysis in insects.

The increase in β-glucosidase activity in esculin-fed females suggests that this enzyme is capable of hydrolyzing hydrophobic substrates. Previous work on insect β-glucosidases [23, 54, 56] has shown the presence of different catalytic sites, one for the disaccharide binding and the other for hydrophobic aglycones. Esculetin *in vitro* effects seems to confirm this suggestion since this aglycone caused an increase in the β-glucosidase activity of males. However, purification and characterization of the enzyme are necessary to confirm this.

Due to the fluorescence of esculin, we were able to visualize the phlebotomines fed on this compound under UV-radiation and quantify its ingestion. Although fluorescence in sucrose/esculin-fed females was statistically higher than males only in samples collected 1-day post-feeding, there is an apparent tendency of higher values in females. Using esculin fluorescence as an indirect measure of ingested sucrose, the amount of sugar ingested seems to be higher in females and it is consistent with the reduction of the average lifespan in esculin-fed flies, due to a higher dose of the toxic compound. Additionally, our data show that methanol can be used as a stabilizing agent, avoiding the chemical and biological hydrolysis of esculin, making methanol a suitable agent to preserve field sand fly samples exposed to sugar baits impregnated with esculin as a monitoring tool.

As expected, esculin was found in the crop, a storage compartment for sugar, migrating successively to the midgut and the rest of the body. This information is similar to the described in *S. frugiperda* larvae [27], where esculin migrates and is slowly absorbed through the gut. The polarity and size of esculin, a molecule containing two aromatic rings and a glycosyl residue, with five exposed hydroxyl groups, may be determinant for its low absorption. The slow absorption of esculin in sand flies might explain the lack of changes in the target enzymatic activities, due to a limited exposition to the ingested compound.

The quantitation of sugar ingested with esculin assumed that this compound is not degraded and fluoresces at the same standard wavelengths. We should consider that these amounts might correspond to a steady-state condition, so they should be considered as minimal amounts ingested in the seven-day period, rather than the total ingestion during the experiment. Daily estimates might clarify this. The presence of esculinase in sand fly tissues is relevant in this context and might have affected the measurements. However, esculetin presents blue fluorescence in alkaline solutions [57], so it was probably measured together with the ingested esculin.

Females ingested higher amounts of sugar and should be more exposed to higher amounts of the toxic compounds esculin/esculetin, which might result in more pronounced physiological effects of the supplements added. However, males after ingesting a 35% smaller amount of beta-glycosides mixed with the sugar meal, presented a reduction in longevity very similar to females. To our knowledge, this is the first quantitative record of the amount of sugar ingested by a sand fly species.

It is interesting to notice the difference between ingested sugar amounts in the conditions tested and blood, which is acquired only by females in values ranging between 0.1–0.6 mg [58]. Blood-fed females are easy to distinguish visually from non-fed. Contrastingly, sugar-fed flies can be identified only with crop or midgut dissection in a magnifying glass or microscope stereoscope, or with the addition of dyes to the sugar meal. Our quantitations are consistent with these observations, showing that the amount of sugar ingested by females is about 7 to 42 times smaller compared to the amount of blood in a single meal.

The *in vitro* exposition of parasites to esculetin only affected *L. infantum* and *L. mexicana* (subgenus *Leishmania*), suggesting that this compound might affect specific metabolic pathways of species in this subgenus. It is interesting that esculetin presented an adverse effect in *Leishmania*, but it seems to be non-harmful (or even beneficial) to the sand fly. Data from the literature have shown *in vivo* anti-*Leishmania* activity of the alkaloid furoquinoline on *L. amazonensis*, a compound also present in plants of the family Rutaceae [7]. It has been suggested that this takes place by the generation of free radicals and DNA topoisomerase I inhibition, which leads to cell death by apoptosis of promastigotes. *In vivo* tests were performed on *L. amazonensis* with another coumarins [(+)-3-(1'-dimethylallyl)-decursinol and (-)-helietin], but its mechanism has not yet been characterized [59]. Another hypothesis is that esculetin could be damaging oxidation reactions necessary for the metabolism of the parasite since it inhibits reactions with oxygen or peroxides [57].

### Amygdalin and mandelonitrile effects

Mandelonitrile was very toxic for males, but its correspondent glycoside (amygdalin) did not affect the ALS. This information suggests that toxicity is hydrolysis-dependent and needs the release of mandelonitrile. Alternatively, mandelonitrile might be detoxified by the insect during the slow release of the group. These data are in agreement with earlier studies showing that insects could avoid the deleterious toxin effects by reducing the hydrolytic activity of specific glycosidases [27].

Females displayed a reduction in ALS when fed on amygdalin or mandelonitrile. However, the toxicity of mandelonitrile was stronger in males. Therefore, it is possible that there are differences in the sugar digestion process between males and females, constituting a sex-related phenotype. Also, these results lead us to question if amygdalin has a direct toxic effect in females or if being hydrolyzed, with the release of hydrocyanic acid in the insect’s gut.

It is important to mention that in our experiments, the concentration of glycosides and aglycones were set in a weight/volume basis, to allow direct comparison with previous results from other groups that studied the effect of these compounds in other insect orders, such as Lepidoptera and Coleoptera. In spite of the use of different molar concentration of aglycones and glycosides, qualitative differences in the effect of these compounds on sand fly physiology were observed, with the general tendency that adult sand flies seem to be more resistant to glycosides than the already studied lepidopteran (*Spodoptera frugiperda*) and coleopteran (*Tenebrio molitor*) species [27]. It is important to consider in this comparison that previous studies focused on the larval stages of these insects, and that the differences might be related to the fact that we exposed adult forms to the toxic glycosides.

The knowledge about the effect of these glycosides on insects is still scarce. In mammals, cyanide toxicity involves the inactivation of respiratory enzymes. However, the concentration of glycosides in plants is not as high as the lethal doses described for mammals. Doses higher than > 3.5 mg/kg are required to produce such toxic effects in mammals. Since the metabolism can neutralize cyanides and convert them into thiocyanates for elimination in the urine, only large amounts of cyanide would overload this pathway [26, 60].

Sand-flies β-glucosidases were expected in the amygdalin hydrolysis process (Fig. 1b, c). However, due to the presence of two glycosidic bonds in the amygdalin molecule, alternative reactions might occur. A first hydrolysis step may produce (i) glucose and prunasin or (ii) gentiobiose and mandelonitrile. Mandelonitrile may also be produced by a second hydrolysis step of prunasin. Then, mandelonitrile may spontaneously produce hydrogen cyanide (HCN). These catalysis processes may be associated with different enzymes, requiring “B” type β-glucosidases specialized in the break of monosaccharides bound to hydrophobic aglycones [23]. In this respect, opposite results for different species of insects have been obtained: larval growth and pupation period of *Diatraea saccharalis* (Lepidoptera: Pyralidae) were significantly affected by orally-administered amygdalin, whereas in *Spodoptera frugiperda* (Lepidoptera: Noctuidae) there was no effect [23].

Another hypothesis, derived from the general mechanism for the catabolism of cyanogenic glucosides, would be the absence of an α-hydroxynitrile lyase that would be responsible for the conversion of α-hydroxynitrile to ketones and HCN compounds (Fig. 1c). However, such enzymes have not yet been characterized in insects [60, 61]. Different studies have shown that there are two enzymes involved in the possible detoxification mechanisms of cyanogenic glycosides: β-cyanoalanine synthase and rhodanase [60, 61]. However, rhodanase activity in insects has not yet been described so that β-cyanoalanine synthase might be performing this role. Further experiments should be carried out to investigate the presence of these enzymes in sand flies.

The average lifespan reduction in adult *L. longipalpis* exposed to amygdalin (females only) and mandelonitrile (both sexes) suggest that these insects might not be adapted to feed on diets containing such compounds. Additional field investigations on the plant-feeding preferences of this species are necessary since amygdalin and mandelonitrile are present in several fruits such as apricots, peaches and plums [25, 26]. Field studies of fruit attractiveness in *P. papatasi* have shown that nectarine, guava, plum and peach are be among its favorite food sources [16]. The development of attractive sugar baits containing plant-derived glycoside compounds, toxic to sand flies, may be considered as a potential novel tool in the control of this vector.

The absence of anti-feeding properties in sand flies clearly shows that mandelonitrile does not affect the ingestion of sugar and that the shortening in ALS previously observed is not derived from food deprivation, but is probably a direct consequence of mandelonitrile toxicity. Besides that, tests performed in confinement showed neither repellence nor attraction activities for mandelonitrile. In this respect, it is likely that mandelonitrile will not affect the contact rate between sand flies and sugar baits containing it.

The observed anti-*Leishmania* effects of mandelonitrile against species from the subgenera *Viannia* and *Leishmania* suggest that the damage or the protection against this aglycone may involve the same metabolic pathways. The mandelonitrile concentrations tested here were lower than those already tested, with a similar effect using 1 mM HCN [62, 63]. The possible mechanism of the anti-*Leishmania* effect of mandelonitrile might be its decomposition with a subsequent release of HCN. Results reported that under anaerobiosis or in the presence of cyanide [62], *Leishmania* motility and proliferation were affected due to the inhibition of energy consumption processes resulting in parasite death. Cyanide blocks the IV complex of the respiratory chain of *Leishmania*, a process that is stopped under anaerobiosis or in HCN presence [62–64]. Further mechanistic studies of mandelonitrile action are required to confirm this hypothesis.

Furthermore, mandelonitrile had a deleterious effect on the *in vivo* development of parasites in the midgut of female sand flies. That was confirmed by two independent techniques, hemocytometer counting and qPCR detection of parasite DNA. Although hemocytometer counting allows visualization of the integrity of the parasite, it is less sensitive than qPCR. Quantification with qPCR confirmed the results obtained by traditional cell counting with increased sensitivity. Some cell counts showed values of zero parasites, leading to “false negative” females, while with the molecular technique *Leishmania* DNA was detected, even at low parasitic loads. These results do not oppose each other. Despite the observation that traditional cell counting resulted in the presence of more negative females than when using qPCR, the deleterious effect of mandelonitrile was statistically significant using both techniques. Additionally, we should also consider that the qPCR might be detecting DNA from unviable or dead parasites. Improvement of the technique may be achieved by detection of parasite mRNA and standardization of an RT-qPCR protocol for infected sand flies. Similar results were obtained in the analysis of plant extracts [12, 13].

HCN has a median lethal concentration in mice (LC50) of 501 ppm (5 min/mouse) [65] and an oral median lethal dose (LD 50) of 3–4 mg/kg [66], while the LC50 of mandelonitrile in mice is 116 mg/kg [67]. The highest amount used in our experiments is equivalent to 100 mg/l of solution. That means that a 350 g mouse would need to ingest more than 441 ml of this solution to cause significant lethality, high above the daily water intake in these animals (approximately 25 ml) [68]. The breakdown of mandelonitrile into hydrocyanic acid is another important factor in choosing mandelonitrile related compounds as possible drug candidates. Moreover, cyanide may be transformed into thiocyanate (SCN^-^) and eliminated in the urine (Fig. 1d) [26]. These detoxification pathways might explain why such high doses of mandelonitrile are required to cause damage in mammals. So the use of mandelonitrile related compounds in applications to control leishmaniasis might be considered in further research projects due to its low toxicity in mammals.

Different compounds of natural origin or synthetic analogous with anti-*Leishmania* activity have been described. Active synthetic compounds have been produced from naphthoquinones [6], chalcones, saponins and alkaloids [7, 69]. Active natural compounds include furoquinolines and coumarins [59]. So far, these molecules have only been studied focusing on their *in vitro* effects against parasites or in mammal infections. *In vitro* assays usually evaluate oxidative stress, loss of mitochondrial membrane potential, modifications in ATP synthesis and cytochrome *c* oxidase and reductase activity, using KCN as a negative control [6, 59, 70, 71]. Future studies should confirm if mandelonitrile has those same effects *in vitro* and during the infection of the vector.

Transmission-blocking attractive sugar baits targeting the parasite in principle would not create selective pressure in field insect populations, being an alternative to the use of chemical insecticides. A problem that should be anticipated is the selection of resistant forms of the parasite. In this case, compounds targeting the promastigote forms that occur only in the sand fly midgut would be strategic, as they would less likely interfere with the resistance status of the amastigotes. Nevertheless, several limitations to the use of this approach in the field may be anticipated, like environmental stability, attractiveness for sand flies in the field and exposure of the baits to non-target insects. However, it should be reinforced that our findings remain as a proof of concept, with the need for adequate subsequential application development. Even if this approach succeeds in future field trials, it must be considered as just one more tool for the management of sand fly populations, to be used in conjunction with the other available strategies. More studies exploring the application of transmission-blocking sugar baits, targeting the *Leishmania* parasites, would be extremely desirable and may strengthen this concept for the control of leishmaniasis or even other vector-borne diseases.

## Conclusions

The number of cases of human leishmaniasis is currently increasing due to several factors. Vector control is one of the strategies for the reduction of transmission of *Leishmania* parasites. Our study showed that some natural glycosides and their aglycones reduce the longevity of the sand fly vector. Additionally, some of these compounds showed anti-*Leishmania* activity, reducing the parasite numbers inside the vector when administered into sugar meals. This concept might be further explored as an environmentally-friendly alternative to vector control and parasite transmission blocking.

## Additional files

Additional file 1: **Table S1.** Esculin hydrolysis activity in different tissues of adult *L. longipalpis* fed on sucrose. **Table S2.** Esculin hydrolysis activity in different tissues of adult male *L. longipalpis* fed on esculin-supplemented sucrose. **Table S3.** Esculin hydrolysis activity in different tissues of adult female *L. longipalpis* fed on esculin-supplemented sucrose. **Table S4.** *In vitro* effect of esculin on trehalase activity of adult male *L. longipalpis*. Tissue homogenates were incubated or not with esculin, and enzyme assays were performed using trehalose as a substrate. **Table S5.** Trehalase activity in adult male *L. longipalpis* after seven days of feeding on sucrose or sucrose with esculin. Sucrose supplemented with esculin 0.1% (w/v). **Table S6.** Trehalase activity in adult female *L. longipalpis* after seven days of feeding with sucrose or sucrose with esculin. Sucrose supplemented with esculin 0.1% (w/v). **Table S7.** *In vitro* effect of esculin on β-glucosidase activities of adult male *L. longipalpis.* Tissue homogenates were incubated with esculin and β-glucosidase was measured using cellobiose as a substrate. **Table S8.** *In vitro* effect of esculin on β-glucosidase activity in adult female *L. longipalpis*. Tissue homogenates were incubated with esculin and β-glucosidase was measured using cellobiose as a substrate. **Table S9.** Effect of supplementation of sucrose diet with esculin on β-glucosidase activities in male *L. longipalpis*. Tissue homogenates were assayed using cellobiose as a substrate. **Table S10.** *In vitro* effect of esculetin on the β-glucosidase activities in female *L. longipalpis*. Tissue homogenates were incubated with esculetin and then assayed using cellobiose as a substrate. (DOCX 27 kb)

Additional file 2: **Figure S1.** Control sample quantification: female sand flies fed only on sucrose. C_t_ means according to parasites numbers per gut. (TIF 124 kb)

Additional file 3: **Figure S2.** Experimental (mandelonitrile) sample quantification: female sand flies fed on sucrose plus mandelonitrile 0.1% (w/v). C_t_ means according to parasite numbers per gut. (TIF 14 kb)

## Abbreviations

ALS: Average lifespan
BOD: Biological oxygen demand
CG: Cyanogenic glycosides
CL: Cutaneous leishmaniasis
CLIOC: Coleção de *Leishmania* do Instituto Oswaldo Cruz
C_t_: Threshold cycle in quantitative PCR
DDT: Dichlorodiphenyltrichloroethane
E64: N-[[trans-eEpoxysuccinyl]]-L-leucine 4-guanidinobutylamide
EDTA: Ethylenediaminetetraacetic acid
MCL: Mucocutaneous leishmaniasis
NTD: Neglected tropical diseases
PAHO: Pan American Health Organization
PBS: Phosphate-buffered saline
PMSF: Phenylmethylsulfonyl fluoride
qPCR: Quantitative polymerase chain reaction
RP49: Ribossomal protein 49
TE: Tris-EDTA
TGO: Tris-glucose-oxidase
U: Unit of enzyme activity
VL: Visceral leishmaniasis
