# Supplementary material for: Transmission blocking sugar baits for the control of Leishmania development inside sand flies using environmentally friendly beta-glycosides and their aglycones
Source: Parasit Vectors. 2018 Nov 30;11:614. doi: 10.1186/s13071-018-3122-z (PMC6271627; doi:10.1186/s13071-018-3122-z)
Supplement: Supplementary file 1 — Table S1. Esculin hydrolysis activity in different tissues of adult L. longipalpis fed on sucrose. Table S2. Esculin hydrolysis activity in different tissues of adult male L. longipalpis fed on esculin-supplemented sucrose. Table S3. Esculin hydrolysis activity in different tissues of adult female L. longipalpis fed on esculin-supplemented sucrose. Table S4. In vitro effect of esculin on trehalase activity of adult male L. longipalpis. Tissue homogenates were incubated or not with esculin, and enzyme assays were performed using trehalose as a substrate. Table S5. Trehalase activity in adult male L. longipalpis after seven days of feeding on sucrose or sucrose with esculin. Sucrose supplemented with esculin 0.1% (w/v). Table S6. Trehalase activity in adult female L. longipalpis after seven days of feeding with sucrose or sucrose with esculin. Sucrose supplemented with esculin 0.1% (w/v). Table S7. In vitro effect of esculin on β-glucosidase activities of adult male L. longipalpis. Tissue homogenates were incubated with esculin and β-glucosidase was measured using cellobiose as a substrate. Table S8. In vitro effect of esculin on β-glucosidase activity in adult female L. longipalpis. Tissue homogenates were incubated with esculin and β-glucosidase was measured using cellobiose as a substrate. Table S9. Effect of supplementation of sucrose diet with esculin on β-glucosidase activities in male L. longipalpis. Tissue homogenates were assayed using cellobiose as a substrate. Table S10. In vitro effect of esculetin on the β-glucosidase activities in female L. longipalpis. Tissue homogenates were incubated with esculetin and then assayed using cellobiose as a substrate. (DOCX 27 kb) [file 13071_2018_3122_MOESM1_ESM.docx]

**Additional file 1**

**Additional file 1: Table S1**. Esculin hydrolysis activity in different tissues of adult *Lutzomyia longipalpis* fed on sucrose. Tissues were homogenized and separated by centrifugation into gut tissue, gut contents, rest of body tissues and rest of body soluble fraction. Results are presented in μU/insect and are the mean and SEM of 13 biological replicates for males and 11 biological replicates for females, with six insects in each sample.

| **Sample** | **Males** | **Females** |
| --- | --- | --- |
| Gut, tissue | 16 ± 9 | 11 ± 7 |
| Gut, contents | 40 ± 10 | 60 ± 10 |
| Rest of body, tissues | 30 ± 10 | ≤ 4 |
| Rest of body, Soluble fraction | 40 ±20 | 50 ± 30 |

**Additional file 1:** **Table S2.** Esculin hydrolysis activity in different tissues of adult male *Lutzomyia longipalpis* fed on esculin-supplemented sucrose. Tissues were homogenized and separated by centrifugation into gut tissue, gut contents, rest of body tissues and rest of body soluble fraction. Results are presented in μU/insect and are the mean and SEM of 5 biological replicates with four insects in each sample. Control flies were fed non-supplemented sucrose.

| **Sample** | **Control**  **(sucrose only)** | **Esculin**  **Supplemented** |
| --- | --- | --- |
| Gut, tissue | ≤ 17 | 20 ± 10 |
| Gut, contents | 20 ± 10 | 0 |
| Rest of body, tissues | 20 ± 10 | 0 |
| Rest of body, Soluble fraction | 20 ± 10 | 30 ± 20 |

**Additional file 1:** **Table S3.** Esculin hydrolysis activity in different tissues of adult female *Lutzomyia longipalpis* fed with esculin-supplemented sucrose. Tissues were homogenized and separated by centrifugation into gut tissue, gut contents, rest of body tissues and rest of body soluble fraction. Results are presented in μU/insect and are the mean and SEM of 5 biological replicates and with four insects in each sample.

| **Sample** | **Control**  **(sucrose only)** | **Esculin**  **Supplemented** |
| --- | --- | --- |
| Gut, tissue | 0 | 0 |
| Gut, contents | 30 ± 10 | 40 ± 20 |
| Rest of body, tissues | 120 ± 60 | 0 |
| Rest of body, Soluble fraction | 70 ± 50 | 40 ± 20 |

**Additional file 1:** **Table S4.** *In vitro* effect of esculin on trehalase activity of adult male *Lutzomyia longipalpis*. Tissue homogenates were incubated or not with esculin*,* and enzyme assays were performed using trehalose as a substrate. Tissues were homogenized and separated by centrifugation into gut tissue, gut contents, carcass tissues and carcass soluble fraction. Results are presented in μU/insect and are the mean and SEM of 4 biological replicates with four insects in each sample.

| **Sample** | **Control**  **(trehalose only)** | **Esculin added to the assay mixture** |
| --- | --- | --- |
| Gut, tissue | 370 ± 60 | 300 ± 100 |
| Gut, contents | 110 ± 10 | 170 ± 40 |
| Rest of body, tissues | 380 ± 40 | 500 ± 100 |
| Rest of body, Soluble fraction | 430 ± 30 | 400 ± 100 |

**Additional file 1:** **Table S5.** Trehalase activity in adult male *Lutzomyia longipalpis* after seven days of feeding with sucrose or sucrose with esculin*.* Sucrose supplemented with esculin 0.1% (w/v)*.* Tissue homogenates were assayed using trehalose as substrate. Tissues were homogenized and separated by centrifugation into gut tissue, gut contents, rest of body tissues and rest of body soluble fraction. Results are presented in μU/insect and are the mean and SEM of 4 biological replicates with five insects in each sample.

| **Sample** | **Control**  **(sucrose only)** | **Esculin**  **Supplemented** |
| --- | --- | --- |
| Gut, tissue | 290 ± 80 | 220 ± 90 |
| Gut, contents | 200 ± 100 | 39 ± 0,4 |
| Rest of body, tissues | 250 ± 60 | 400 ± 100 |
| Rest of body, Soluble fraction | 430 ± 70 | 400 ± 100 |

**Additional file 1:** **Table S6.** Trehalase activity in adult female *Lutzomyia longipalpis* after seven days of feeding with sucrose or sucrose with esculin. Sucrose supplemented with esculin 0.1% (w/v)*.* Tissue homogenates were assayed using trehalose as substrate. Tissues were homogenized and separated by centrifugation into gut tissue, gut contents, rest of body tissues and rest of body soluble fraction. Results are presented in μU/insect and are the mean and SEM of 4 biological replicates with five insects in each sample.

| **Sample** | **Control**  **(sucrose only)** | **Esculin**  **Supplemented** |
| --- | --- | --- |
| Gut, tissue | 220 ± 40 | 200 ± 70 |
| Gut, contents | 50 ± 10 | 130 ± 90 |
| Rest of body, tissues | 400 ± 100 | 570 ± 90 |
| Rest of body, Soluble fraction | 450 ± 80 | 600 ± 90 |

**Additional file 1:** **Table S7.** *In vitro* effect of esculin on β-Glycosidase activities of adult male *Lutzomyia longipalpis.* Tissue homogenates were incubated with esculin and β-Glycosidase was measured using cellobiose as a substrate. Tissues were homogenized and separated by centrifugation into gut tissue, gut contents, rest of body tissues and rest of body soluble fraction. Results are presented in μU/insect and are the mean and SEM of 5 biological replicates with 20 insects in each sample.

| **Tissue, Fraction** | **Control**  **(cellobiose only)** | **Esculin added to the assay mixture** |
| --- | --- | --- |
| Gut, tissue | 4 ± 2 | 1 ± 1 |
| Gut, contents | 16 ± 4 | 14 ± 7 |
| Rest of body, tissues | 7 ± 3 | 3 ± 1 |
| Rest of body, Soluble fraction | 6 ± 2 | 3 ± 2 |

**Additional file 1:** **Table S8.** *In vitro* effect of esculin on β-Glycosidase activity in adult female *Lutzomyia longipalpis.* Tissue homogenates were incubated with esculin and β-glycosidase was measured using cellobiose as a substrate. Tissues were homogenized and separated by centrifugation into gut tissue, gut contents, rest of body tissues and rest of body soluble fraction. Results are presented in μU/insect and are the mean and SEM of 5 biological replicates with 20 insects in each sample.

| **Tissue, Fraction** | **Control**  **(cellobiose only)** | **Esculin added to the assay mixture** |
| --- | --- | --- |
| Gut, tissue | 2 ±1 | 2 ± 1 |
| Gut, contents | 36 ± 18 | 23 ± 8 |
| Rest of body, tissues | 5 ± 2 | 4 ± 3 |
| Rest of body, Soluble fraction | 21 ± 12 | 4 ± 1 |

**Additional file 1:** **Table S9.** Effect of supplementation of sucrose diet with esculin on β-glucosidase activity in male *Lutzomyia longipalpis.* Tissue homogenates were assayed using cellobiose as a substrate. Tissues were homogenized and separated by centrifugation into gut tissue, gut contents, rest of body tissues and rest of body soluble fraction. Results are presented in μU/insect and are the mean and SEM of 5 biological replicates with ten insects in each sample.

| **Tissue, Fraction** | **Control**  **(sucrose only)** | **Esculin**  **supplemented** |
| --- | --- | --- |
| Gut, tissue | 0.4 ± 0.3 | 0.9 ± 0.4 |
| Gut, contents | 4.6 ± 0.2 | 4 ± 2 |
| Rest of body, tissues | 0 | 2 ± 1 |
| Rest of body, Soluble fraction | 3.4 ± 0.7 | 1.9 ± 0.7 |

**Additional file 1:** **Table S10.** *In vitro* effect of Esculetin in the β-Glycosidase activity in female *Lutzomyia longipalpis.* Tissue homogenates were incubated with esculetin and then assayed using cellobiose as substrate. Tissues were homogenized and separated by centrifugation into gut tissue, gut contents, rest of body tissues and rest of body soluble fraction. Results are presented in μU/insect and are the mean and SEM of 4 biological replicates with 20 insects in each sample.

| **Tissue, Fraction** | **Control**  **(cellobiose only)** | **Esculetin added to the assay mixture** |
| --- | --- | --- |
| Gut, tissue | 5.9 ± 0.8 | 4 ± 2 |
| Gut, contents | 27 ± 3 | 23 ± 9 |
| Rest of body, tissues | 6.3 ± 0.6 | 7.8 ± 2 |
| Rest of body, Soluble fraction | 4 ± 2 | 2.5 ± 1.2 |
