# Supplementary figures and images for: Transmission blocking sugar baits for the control of Leishmania development inside sand flies using environmentally friendly beta-glycosides and their aglycones
Source: Parasit Vectors. 2018 Nov 30;11:614. doi: 10.1186/s13071-018-3122-z (PMC6271627; doi:10.1186/s13071-018-3122-z)

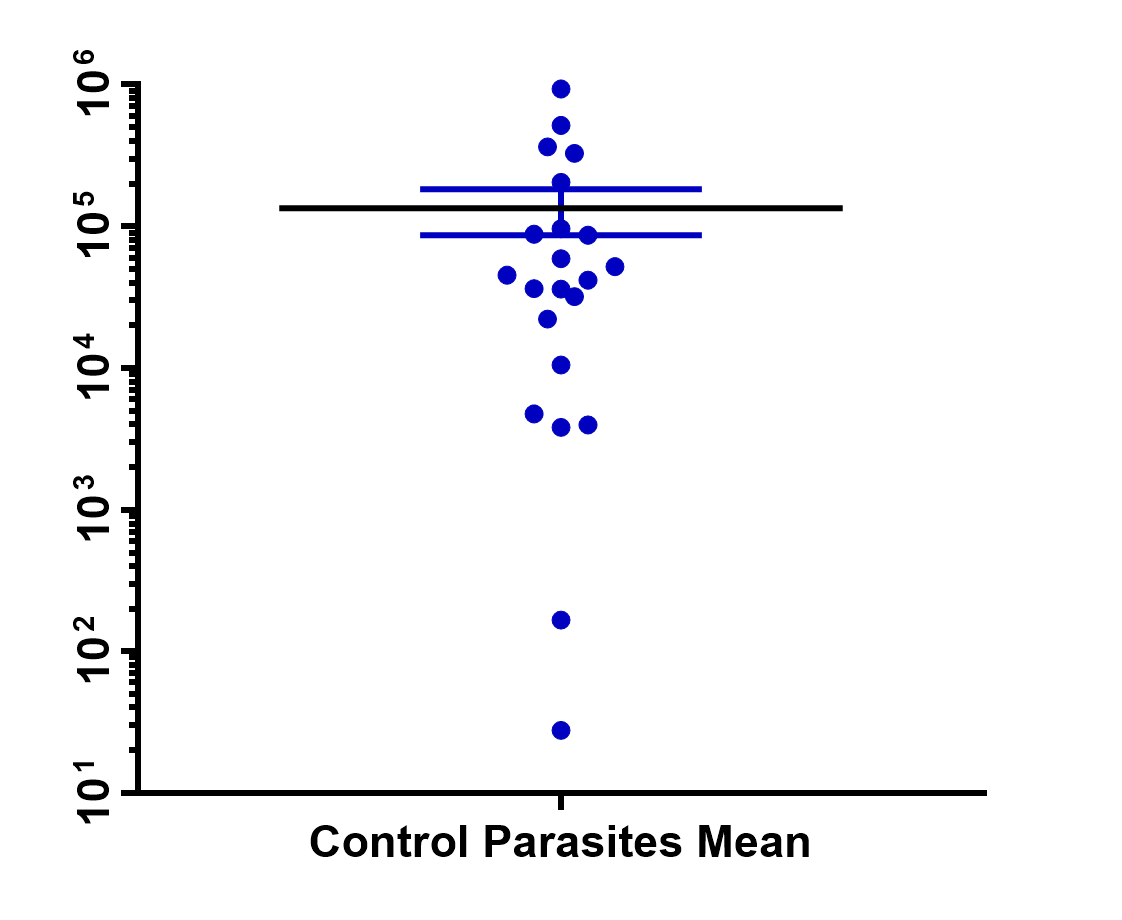

Supplement: Supplementary file 2 — Figure S1. Control sample quantification: female sand flies fed only on sucrose. Ct means according to parasites numbers per gut. (TIF 124 kb) [file 13071_2018_3122_MOESM2_ESM.tif]
